# Electronic and Inertial Effects of Methylation on Excited-State Hydrogen Transfer

**DOI:** 10.1021/acs.jpca.5c07439

**Published:** 2026-01-27

**Authors:** Pratip Chakraborty, Rafael C. Couto, Nanna H. List

**Affiliations:** † Department of Chemistry, 6106 KTH Royal Institute of Technology, Stockholm SE 10044, Sweden; ‡ School of Chemistry, 7655 University of Birmingham, Birmingham B15 2TT, United Kingdom

## Abstract

Excited-state intramolecular hydrogen transfer (ESIHT) is among the fastest chemical reactions and is a key design element in photoprotective molecules and functional chromophores. Despite the apparent simplicity of the symmetric HO–C=C–C=O ESIHT prototype, its multifunctional nature enables competing nonradiative decay channels, including C=C torsional motion. Here, we compare malonaldehyde (MA), the minimal motif, with its methylated derivative acetylacetone (AcAc) to investigate how electronic and inertial effects of methylation shape the ultrafast dynamics initiated on S_2_(ππ*). XMS-CASPT2 nonadiabatic dynamics on the singlet manifold reveal bond-length alternation that drives the wavepacket toward the H-transfer intersection seam rather than undergoing torsional motion directly out of the Franck–Condon region. Methylation destabilizes the S_1_(nπ*) state, reducing the S_2_/S_1_-energy gap and enhancing the asymmetry of the H-transfer intersection seam. As a result, S_2_/S_1_-decay precedes H-transfer, which mostly takes place only after the population arrives on S_1_. Moreover, the methyl groups in AcAc introduce an inertial mismatch between the central methine hydrogen and the terminal methyl groups, which gives rise to two distinct behaviors on S_1_: (i) an early ballistic rise in ground-state population within ∼75 fs via twist-pyramidalized geometries akin to the behavior of α,β-enones and (ii) a slower repopulation through torsional motion, with the majority of the population remaining near the planar S_1_-minimum. In contrast, MA displays no ballistic channel. Our results for AcAc are consistent with recent time-resolved photoelectron spectroscopy, confirming the ultrafast S_2_-lifetime. We propose extending such experiments into the X-ray regime, where the evolution of the oxygen 1s binding energies offers direct, site-specific sensitivity to the H-transfer-mediated motion governing the early decay.

## Graphical abstract

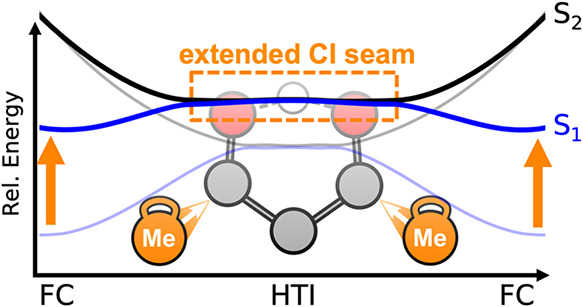

## 1 Introduction

Excited-state intramolecular hydrogen transfer (ESIHT) is among the fastest known chemical reactions, typically unfolding within tens of femtoseconds.^1–5^ It plays a key role in diverse biological processes^1,2^ and serves as a photofunctional unit in a range of light-driven technologies.^6–9^ Despite its apparent structural simplicity, the HO–C=C–C=O enolone motif underlying symmetric ESIHT supports competing nonradiative decay channels, including H-transfer and C=C torsional motion. The latter pathway is well-established in α,β-enones (C=C–C=O), where excitation of the S_2_(ππ*) state weakens the double bond and promotes ultrafast torsion-mediated decay.^10–12^

Chemical substitution provides a powerful knob to tune photochemical reactivity.^13–22^ Substituents influence dynamics through two mechanisms:^23^ (i) electronic effects, which modify potential-energy surfaces (PESs) by stabilizing or destabilizing regions of particular electronic character, and (ii) inertial effects, which alter specific nuclear modes, changing the direction and velocity of the nuclear wavepacket. Methylation is an important example with relevance also in biological settings (see e.g., refs 24,25 ). While methyl groups are traditionally considered inertial substituents,^16,17^ they also exert weak electron-donating effects that can stabilize/destabilize charge-polarized configurations depending on their position. Methyl substitution has, for example, been shown to significantly affect the excited-state dynamics of unsaturated hydrocarbons, allenes, carbonyls,^12,18,26^ but its role in enolone systems has not been systematically investigated with high-level nonadiabatic dynamics.

Malonaldehyde (MA) is formally the smallest (symmetric) β-diketone, but in the gas phase it predominantly exists as the enolone tautomer stabilized by electronic conjugation and intramolecular hydrogen bonding (Figure 1).^27–35^ This renders MA the minimal HO–C=C–C=O system and hence a prototype for ESIHT.^36^ Acetylacetone (AcAc) retains the enolone tautomer,^37^ but the methyl groups lower symmetry and introduce both electronic and inertial perturbations. Both molecules share a basic electronic structure with a dark S_1_(nπ*) state lying below the bright S_2_(ππ*) state.^38,39^ However, while gas-phase MA is unstable at room temperature and thus experimentally challenging,^40^ AcAc is stable and has been the subject of extensive time-resolved spectroscopic studies.

**Figure 1:**
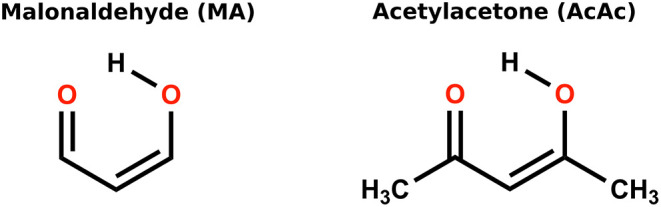
Molecular structures of the enolone tautomer of malonaldehyde (MA) and acetylacetone (AcAc).

CASSCF-based nonadiabatic dynamics simulations have reported a competition between H-transfer and torsion in MA and AcAc. For MA, the wavepacket was found to decay predominantly via a higher-lying H-transfer intersection, with subsequent relaxation on S_1_ proceeding through torsional motion.^41–43^ For AcAc, H-transfer was also found to be the dominant S_2_/S_1_-decay pathway, with torsion accounting for ∼25% of the population transfer.^44^ Došlić and co-workers later compared MA and AcAc dynamics at the CASSCF and ADC(2) levels,^45,46^ focusing mainly on the fate of the S_1_ wavepacket and differences in triplet quantum yields. These studies provided important first comparative insights, yet further investigation is warranted to overcome the limitations of CASSCF and ADC(2) for excited-state dynamics in carbonyl-containing systems: CASSCF neglects dynamical electron correlation, leading to blue-shifted excitation energies and overestimated S_2_/S_1_-gaps, while ADC(2) is prone to artificial S_1_/S_0_-crossings that mediate premature S_1_(nπ*)-decay.^47^ Very recently, XMS-CASPT2 dynamics were reported for AcAc alone,^48^ offering a more reliable description of the electronic structure but leaving the role of methylation unresolved.

The early dynamics of AcAc in the gas phase following photoexcitation (near 260 nm) to the S_2_(ππ*) have also been probed experimentally by a variety of techniques, including ultrafast electron diffraction,^49,50^ femtosecond pump–probe photoionization with electron and ion detection,^51^ photoelectron spectroscopy,^46,48,52^ and transient carbon *K*-edge X-ray absorption.^53^ Recent experiments indicate that AcAc undergoes ultrafast internal conversion to the S_1_(nπ*) state within the first 20 fs, followed by intersystem crossing to T_1_ with a time constant of ∼1.5 ps. Longer time scale dynamics (tens to a few hundreds of picoseconds) have been assigned to the relaxation of the *T*_1_ state and intersystem crossing to the ground state. Detailed characterization of the photoproducts (after photoexcitation at 248, 266, or 280 nm) suggests both Norrish type-I pathways from the T_1_ state and phototautomerization followed by dissociation from the vibrationally hot ground state.^54,55^ Despite these advances, the role of methylation in shaping ultrafast dynamics in enolones, particularly the competition between the photochemistry of different functional units, remains unresolved, and no high-level comparative dynamics of MA and AcAc has yet been reported.

To address this gap, we report trajectory surface hopping simulations of MA and AcAc at the XMS-CASPT2 level, disentangling the electronic and inertial effects of methylation and establishing how these factors modulate the earliest internal conversion dynamics. To capture the influence of low-frequency methyl rotations and possible H-tunneling on the ground state, initial conditions for the nonadiabatic dynamics simulations were sampled from quantum-thermostat *ab initio* molecular dynamics^56^ rather than from a harmonic Wigner distribution. We find that S_2_/S_1_-decay in both systems proceeds exclusively via H-transfer-mediated dynamics, with no evidence for the torsion-mediated pathway. While methylation leaves the initial decay largely unchanged, it imparts a more pronounced inertial influence on the ensuing S_1_ dynamics, leading to different extents of ground-state recovery.

## 2 Computational Details

This section outlines the computational setup used in this work. An overview of the key parameters for initial-condition generation, electronic-structure method, and nonadiabatic dynamics simulations is provided in Section S1.

### 2.1 Electronic-Structure Level and Benchmark

We described the electronic structures of MA and AcAc using extended multistate complete active-space second-order perturbation theory (XMS-CASPT2)^57–59^ based on a state-averaged complete active-space self-consistent field reference, as implemented in BAGEL 1.2.2.^60,61^ Initial-condition sampling was handled separately (see below). For the singlet manifold, we included the three lowest states (with equal weights) in the state-averaging (SA), whereas a two-state average was used for the triplet manifold. To serve as a reference for benchmark, we computed critical points and their energies along interpolated paths with a larger active space comprised of 14 electrons in 12 orbitals (see Figure S2 of the Supporting Information for an illustration of the orbitals) using the multistate multireference (MSMR)^62^ contraction scheme with frozen core and a real shift of 0.3 *E*_*h*_ (no IPEA shift). This electronic-structure level is denoted as SA3­(SA2 for triplets)-XMS­(Re=0.3)-CASPT2­(14,12) in the following. Based on a favorable agreement with this reference, we selected a smaller active space of 10 electrons in 8 orbitals with a single-state single-reference (SSSR)^62^ contraction scheme as a cost-effective alternative for the production calculations (UV photoabsorption and nonadiabatic dynamics simulations). Specifically, we used SA3-XMS­(Im=0.3)-CASPT2­(10,8) with a frozen core and an imaginary level shift of 0.3 *E*_*h*_ (no IPEA shift). All calculations used the cc-pVDZ basis set together with the cc-pVDZ-jkfit density-fitting basis. Benchmark results are provided in Section S2 together with a discussion of active-space stability in Section S3.

### 2.2 Initial-Condition Sampling

The methyl groups in AcAc complicate initial-condition (IC) sampling using a harmonic Wigner distribution. In particular, the low-frequency torsional mode associated with ketonic methyl rotation is poorly described by a linear approximation, leading to artificially long C–H bonds and potential active-space instabilities. While excluding such modes has been used when they are deemed dynamically irrelevant,^22,63^ this may not be appropriate here, as methyl orientation influences which regions of the intersection seam are accessed (see below). Moreover, the shallow barriers for methyl rotation give rise to multiple distinct ground-state minima that are not captured by Wigner sampling around the global staggered minimum. To address these limitations, we employed quantum-thermostat *ab initio* molecular dynamics (QT-AIMD^64,65^ ) to generate ICs, as recently proposed by Prlj et al.^56,66^

QT-AIMD was performed at the density-functional theory^67,68^ level with the B3LYP^69–72^ exchange–correlation functional using the D3-BJ^73,74^ dispersion correction and the 6–31G­(d,p)^75–77^ basis set. The dynamics were initiated from the corresponding optimized geometries (AcAc with methyl groups in a staggered conformation), with velocities drawn from a Boltzmann distribution. We used a time step of ∼0.48 fs (20 au) and QT parameters (GLE drift and diffusion matrices A and C) from the GLE4MD database,^78^ corresponding to a temperature of 298.15 K, Ns = 6, and ℏω_max_/*k*_B_ *T* = 20 (strong-coupling regime). For this temperature, ω_max_ = 4114.5 cm^–1^, which exceeds the highest-frequency normal mode of both MA and AcAc. We determined the equilibration time of the trajectories by monitoring the convergence of the average kinetic energy.^56^ From the thermalized trajectories, we extracted 1890 and 1784 samples for MA and AcAc, respectively. Snapshots were sampled in ∼120 fs intervals (lowest frequency ∼283 cm^–1^) and ∼710 fs intervals (lowest-frequency methyl rotation is ∼48 cm^–1^) for AcAc. All QT-AIMD simulations were carried out using the ABIN code^79^ interfaced with the TeraChem program^80–83^ for the electronic-structure calculations.

We also generated 5000 initial conditions for both molecules from a thermal harmonic Wigner distribution at 298.15 K using B3LYP­(D3-BJ)/6–31G­(d,p)-optimized geometries and normal modes. A comparison of the results of the two IC sampling methods and the advantages of QT-AIMD sampling for AcAc is provided in Section S4.

### 2.3 Nonadiabatic Molecular Dynamics

ICs for the nonadiabatic dynamics simulations were selected to mimic 266 nm (4.661 eV) photoexcitation of the first absorption band of AcAc (experimental maximum at 4.716 eV in the gas phase^39^). Vertical excitation energies and oscillator strengths were computed for all QT-AIMD sampled geometries at the SA3-XMS­(Im=0.3)-CASPT2­(10,8)/cc-pVDZ (SSSR) level. To simulate the absorption spectrum, the resulting stick spectra were convolved with a Gaussian line shape (FWHM = 0.24 eV). For AcAc, the spectrum was uniformly shifted by +0.096 eV to align the first absorption maximum with the experimental data (Figure S11). The same broadening and energy shift were applied for MA. To approximate the finite bandwidth of a typical pump pulse, initial conditions were selected from a 0.1 eV window around the pump energy (i.e., 4.661 ± 0.05 eV after applying the uniform shift). Within this window, 265 ICs were selected for AcAc, with 250 and 15 ICs having S_2_ and S_1_ as the bright state, respectively. For MA, 256 ICs were selected, of which 255 had S_2_ and one had S_1_ as the bright state.

The nonadiabatic dynamics simulations were carried out using SHARC 3.0 (Surface Hopping including ARbitrary Couplings)^84^ interfaced with BAGEL 1.2.2.^60,61^ We performed trajectory surface hopping simulations on SA3-XMS­(Im=0.3)-CASPT2­(10,8)/cc-pVDZ full-dimensional potential-energy surfaces (PESs) calculated in the molecular Coulomb Hamiltonian (MCH) representation for a duration of 200 fs. The fewest-switches surface hopping (FSSH)^85^ algorithm was used to take into account nonadiabatic events among the S_2_, S_1_, and S_0_ states. We employed an energy-based decoherence correction using the recommended α = 0.1 *E*_*h*_ value.^86^ Given the short simulation time, we did not apply any corrections to zero-point leakage. The velocity Verlet algorithm was employed to integrate the nuclear equations of motion with a time step of 0.5 fs, while the electronic wave function was propagated with a time step of 0.02 fs. To conserve the total energy after a successful hop, the kinetic energy was adjusted by rescaling the momentum along the nonadiabatic coupling vector, while the momentum direction was left unaltered when a frustrated hop was encountered. Energy conservation was monitored for all trajectories. The reader is referred to Section S3 for details on active-space stability, discarded trajectories, and an analysis of their influence on populations and geometric distributions.

### 2.4 X-ray Photoelectron Spectroscopy Calculations

To assess whether oxygen *K*-edge X-ray photoelectron spectroscopy (XPS) can resolve signatures of H-transfer, we computed the oxygen 1s core–electron binding energies along selected paths and representative trajectories using the extended multistate restricted active-space second-order perturbation theory (XMS-RASPT2).^87^ The five lowest doublet states were included in the state-averaging of the core-ionized states, considering every 1 fs along the selected trajectories. The core-ionized states at each oxygen *K*-edge were computed separately and subsequently combined in the final spectrum. This approach was used due to active-space instabilities when the hydrogen was transferred between the two oxygens, causing the absence of one of the signals. Accordingly, one oxygen 1s-orbital was placed in the RAS1 space (with a one-hole constraint), while the RAS2 space contained the orbitals corresponding to the valence CAS­(14,12), i.e., XMS-RASPT2­(11,1,0;1,12,0) using the notation (*n*, *l*, *m i*, *j*, *k*), where *n* is the number of active electrons, *l* is the maximum numbers of holes in RAS1, and *m* is the maximum number of electrons allowed in RAS3, while *i*, *j*, *k* are the number of active orbitals in RAS1, RAS2, and RAS3, respectively. This larger valence space was needed for the computation of core-ionized states, as instabilities were otherwise observed at distorted geometries. The cc-pVDZ basis set was employed together with an imaginary shift of 0.3 *E*_*h*_ to avoid intruder states (no IPEA shift). The highly excited-state procedure^88^ was used to target the oxygen *K*-edge, and Cholesky decomposition of the two-electron repulsion integrals was employed.^89^ While Dyson norms are often used to approximate photoionization intensities, the similar shapes of the localized 1s-core orbitals can render pre-edge XPS intensities at a given *K*-edge largely comparable.^90^ While assuming stoichiometric ratios is not always valid in XPS,^91^ both experiment and theory support that this is a reasonable approximation in AcAc: the steady-state oxygen *K*-edge XPS spectrum shows ketonic and enolic features of similar intensities,^92,93^ and a restricted active-space state interaction (RASSI^94^) calculation at the Franck–Condon (FC) point confirms a near-unity intensity ratio of 0.93 (based on Dyson norms of 0.56 and 0.60, respectively). For this reason, we assumed uniform intensities for the two oxygen sites in our time-resolved XPS (TRXPS) analysis. All XMS-RASPT2 calculations were performed using OpenMolcas 24.06.^95–97^

## 3 Results and Discussion

We first analyze static potential-energy trends along interpolated paths in AcAc and assess the effects of methylation by comparison with MA. We then examine the time-resolved FSSH dynamics and the resulting oxygen *K*-edge TRXPS signatures of H-transfer. The key reaction coordinates are bond-length alternation (BLA), H-transfer (HT), chelate-ring contraction/expansion, as quantified by the sum-of-angles (SOA) coordinate, pyramidalization of the central methine carbon (PyrC), and C=C torsion. The definitions of these geometric parameters can be found in Section S1.

### 3.1 Signposts on the Potential-Energy Landscapes

Figure 2 presents the XMS-CASPT2­(14,12) potential-energy curves of the lowest singlet and triplet states of AcAc, computed along geodesic interpolated pathways^98^ connecting minima and minimum-energy conical intersections (MECIs). Geometric parameters and relative energies of all critical points are compiled in Table S2, and a brief comparison to experimental and past theoretical results at the FC point can be found in Section S2.

**Figure 2:**
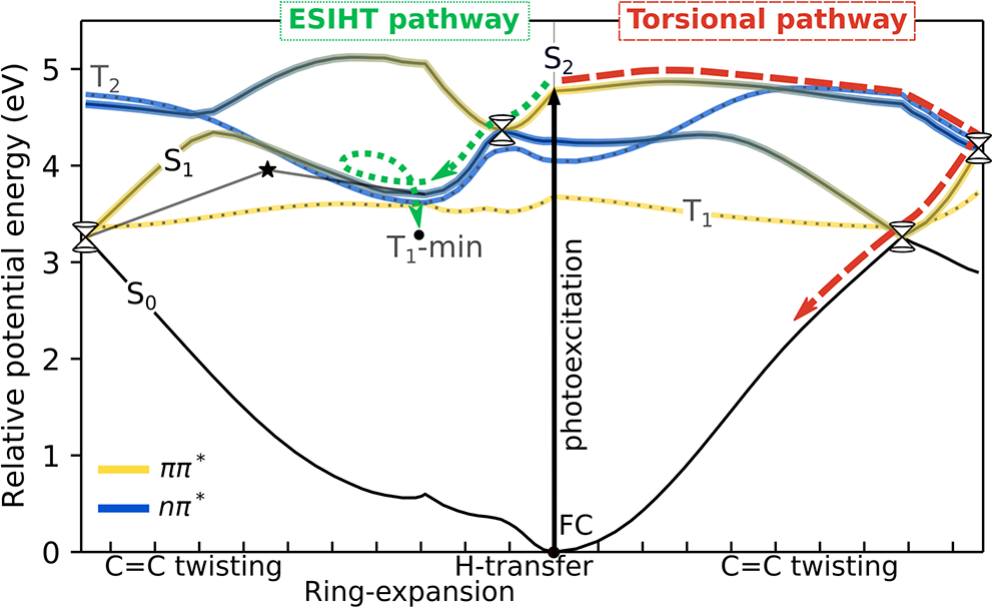
Ground- and valence-excited potential-energy curves for AcAc along geodesic interpolated pathways between minima and MECIs, indicating possible deactivation pathways upon S_2_(ππ*) photoexcitation. The *x*-axis is given in mass-weighted distance 1 Å (·amu^1/2^/tick), considering all atoms except methyl hydrogens. This choice avoids ambiguity arising from different methyl orientations and emphasizes the heavy-atom framework governing the decay pathways. The effect of methyl orientation is discussed in Figure 3. The electronic character along the curves is marked in yellow: ππ* and blue: nπ*, as gauged by the nature of the dominant configuration. The torsional displacement proceeds near an avoided crossing with a concomitant interchange of the characters of S_2_ and S_1_. The asterisk indicates the transition state on S_1_. Critical points were obtained at the SA3­(or SA2)-XMS­(Re=0.3)-CASPT2­(14,12)/cc-pVDZ (MSMR) level of theory with corresponding geometries visualized in Figure S5.

It should be noted that the ground-state PES of AcAc features several shallow minima due to low barriers to intramolecular H-transfer and methyl rotation.^99–106^ This flatness makes the determination of the true ground-state vibrational wave function challenging and still an open question (see brief discussion in Section S4.2). When the zero-point energy is neglected, the staggered conformer is consistently predicted to be the global S_0_-minimum. This is also the case at the present XMS-CASPT2 level of theory.

Following photoexcitation to the bright S_2_(ππ*) state, two distinct deactivation channels leading to S_1_ become accessible, as indicated by the curly arrows in Figure 2. Along the ESIHT pathway (dotted arrow), AcAc can funnel from S_2_ to S_1_ via a planar H-transfer intersection (HTI) seam. From there, the molecule can relax toward a planar, ring-expanded S_1_-minimum. Returning to the ground state from this geometry requires overcoming a torsional barrier of 0.25–0.65 eV (the lower value is the true barrier, whereas the upper bound is from the interpolated path, assuming the dynamics to be sufficiently fast that other degrees of freedom do not have time to relax) due to an avoided crossing with the S_2_ state. Alternatively, and as suggested by some earlier studies,^46,48,53,55^ the system can undergo intersystem crossing (ISC) enabled by the *T*_1_(ππ*) and *T*_2_(nπ*) states being almost degenerate with the S_1_ state near its minimum and El-Sayed allowed.^107^ While the energy profile of the T_2_(nπ*) state displays strong geometry-dependence (approximately following the singlet nπ* diabat), the *T*_1_(ππ*) surface remains relatively flat over the explored configuration space with its minimum at a C=C twisted geometry (*T*_1_-min in Figure 2). We note that the ordering and character of the triplets near the S_1_-minimum are method-dependent^46,48^ and that even small methodological differences can shift their relative energies and the corresponding triplet intersection seam relative to S_1_.

The second pathway (dashed arrow) involves torsional motion, as facilitated by the weakening of the C=C bond in the S_2_(ππ*) state. This leads to a twisted S_2_/S_1_-MECI (S_2_/S_1_-Tw), located ∼0.1 eV below the minimum of the HTI seam. Torsional motion significantly destabilizes the ground state, bringing the three lowest singlet states in energetic proximity (nearby S_1_/S_0_-Tw). As a result, torsion may mediate efficient internal conversion from S_2_ to S_0_ via S_1_, as indicated by some previous CASSCF-based studies.^41,42,44^ The interpolated energy profile suggests that torsional motion proceeds over a small barrier and is accompanied by a change in the character of the electronic state. The corresponding picture for MA is overall similar to that of AcAc, as summarized in Figure S3 and Table S4 (see also Figure 2 in ref 108). Since the HTI seam lies geometrically closer and downhill from the FC region, it is likely the primary pathway for nonradiative decay to S_1_. However, high-level nonadiabatic dynamics incorporating inertial effects are necessary to map out the potential pathway competition. Before reaching that point, we focus on how methylation modifies the static trends.

#### 3.1.1 Methylation Promotes Early Access to the HTI Seam via S_1_-Destabilization

Figure 3 compares the XMS-CASPT2­(14,12) energy profiles of the singlet states in MA (dashed lines) and AcAc (solid lines) along the interpolated H-transfer path, which connects the FC region to the symmetric HTI geometry and back. Key structural changes along this path, including BLA and chelate-ring contraction (SOA), are shown alongside the energy profiles.

**Figure 3:**
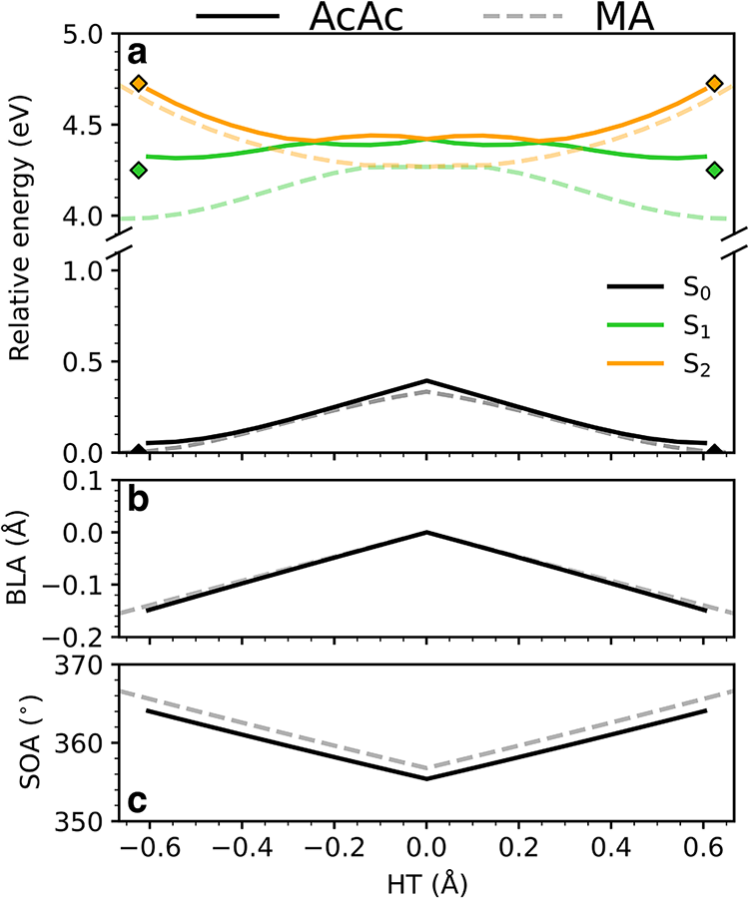
Energy profiles along the H-transfer pathway (linear interpolation from the FC region through the symmetric S_2_/S_1_–HTI to the H-transferred FC region) together with key geometric parameters as obtained at the SA3-XMS­(Re=0.3)-CASPT2­(14,12)/cc-pVDZ (MSMR) level. (a) S_0_, S_1_, and S_2_ energies for MA (dashed) and AcAc (solid) along the H-transfer coordinate. Energies are reported relative to their respective S_0_-minima, and the broken *y*-axis is used for magnification. The H-transfer pathway is characterized by (b) BLA equalization and (c) chelate-ring contraction, as quantified by the SOA coordinate. In the case of AcAc, the eclipsed-up (E_up_) methyl-group conformations have been used. As a result, the S_0_-energy at HT = −0.6 Å is not exactly zero. The E_up_ conformation exerts the strongest electron-donating effects of the methyl group, and hence displays the smallest S_2_/S_1_-gap in the FC region. For AcAc, the energies at the staggered S_0_-minimum (global minimum) are indicated by diamonds.

The energies of the bright S_2_(ππ*) state are nearly identical for MA and AcAc. On the other hand, the S_2_/S_1_-energy gap is ∼0.3–0.4 eV smaller in AcAc. This difference is caused by the weak electron-donating character of the methyl groups, which destabilize the S_1_(nπ*) state accordingly (compare green dashed and solid lines). This electronic effect places AcAc as an intermediate case between MA and canonical ESIHT systems, such as methyl salicylate,^109,110^ for which the S_1_ state is the bright ππ* state. The extent of the methyl-induced destabilization depends on the orientation of the methyl groups: the AcAc profile in Figure 3 corresponds to the eclipsed-up conformer (diamond markers indicate the energies for the ground-state staggered conformer), which shows the largest electronic effect due to increased C–H hyperconjugation.^111^ The size of the S_2_/S_1_-gap also depends on the theory level (here, considering variations in the XMS-CASPT2 setup). With SA3-XMS-CASPT2­(14,12), the energy gaps are ∼0.9 eV for MA and ∼0.5 eV for AcAc. The latter value is consistent with available experimental UV-absorption data,^39^ which is about 0.2 eV smaller than the gap reported in electron energy loss spectroscopy.^112^ The gaps decrease by ∼0.1 eV with the reduced (10,8) active space employed in our dynamics simulations, mainly due to an increase in the S_1_ energy. Accordingly, our dynamic setup may slightly overestimate the rate of S_2_/S_1_-decay. Including five states in the state-averaging would reduce the gap by another ∼0.1 eV and would therefore offer a poorer description near the FC region (see further discussion on the active space in Section S3).

Besides their effect on the FC region, the methyl groups also influence the HTI intersection seam. As shown in Figure 3, methylation extends the accessible configurational space of the seam and introduces pronounced asymmetry along the H-transfer coordinate. In AcAc, the seam is flat, and the MECI is substantially displaced from the *C*_2*v*_-symmetric HTI geometry (HT = ±0.35 Å vs 0 Å). As a result, the symmetric HTI acts as a shallow transition structure on the seam, connecting two equivalent asymmetric HTIs (compare asym-HTI-Eup and sym-HTI-E_up_ in Table S2). In MA, the same qualitative picture holds; however, the degree of asymmetry is reduced. Although the asymmetric structure is also slightly more stable in MA, it is geometrically closer to the symmetric counterpart (HT = ±0.23 Å vs 0 Å, see Table S4). Taken together, this static analysis suggests that methylation enhances the asymmetry of the HTI seam through the destabilization of the S_1_(nπ*) state, shifting the low-energy access to the seam away from the symmetric geometry and potentially facilitating earlier access to S_1_ along the H-transfer coordinate.

### 3.2 Ultrafast Internal Conversion Dynamics

Having established the methylation effects on the potential-energy landscape, we now turn to the dynamically accessed internal conversion pathways and inertial implications of methylation; we performed decoherence-corrected FSSH simulations initiated on the bright ππ* state of both MA and AcAc. Initial conditions were sampled using QT-AIMD and selected within a ±0.05 eV window around 4.661 eV to mimic a 266 nm pump pulse targeting the red side of the lowest absorption band (see Figure S11).

The resulting population dynamics during the first 200 fs following photoexcitation are shown in Figure 4. Although MA exhibits a ∼0.3 eV larger S_2_/S_1_-energy gap in the FC region than AcAc, this translates into only a slightly slower ultrafast S_2_/S_1_-decay (by a few femtoseconds). For both molecules, most of the S_2_ population transfers to S_1_ within 20 fs. A small fraction (∼5–10%) is transiently trapped on S_2_, leading to a population plateau in the 20–60 fs time window (see also Figure S18). The repopulation of the ground state (or the growth and decay of S_1_) is more distinct in the two systems. For AcAc, the repopulation occurs in two stages: an initial (∼15%) faster repopulation within 25–75 fs, followed by a slower transfer reaching a S_0_ population of ∼25% (∼20% when all trajectories are included; see discussion in Section S3) at the end of the 200 fs simulations. For MA, the initial transfer stage is essentially absent and only the slower growth remains, resulting in about half the ground-state repopulation within the 200 fs simulation time compared to AcAc. The underlying mechanisms and differences between the two molecules will be explored next.

**Figure 4:**
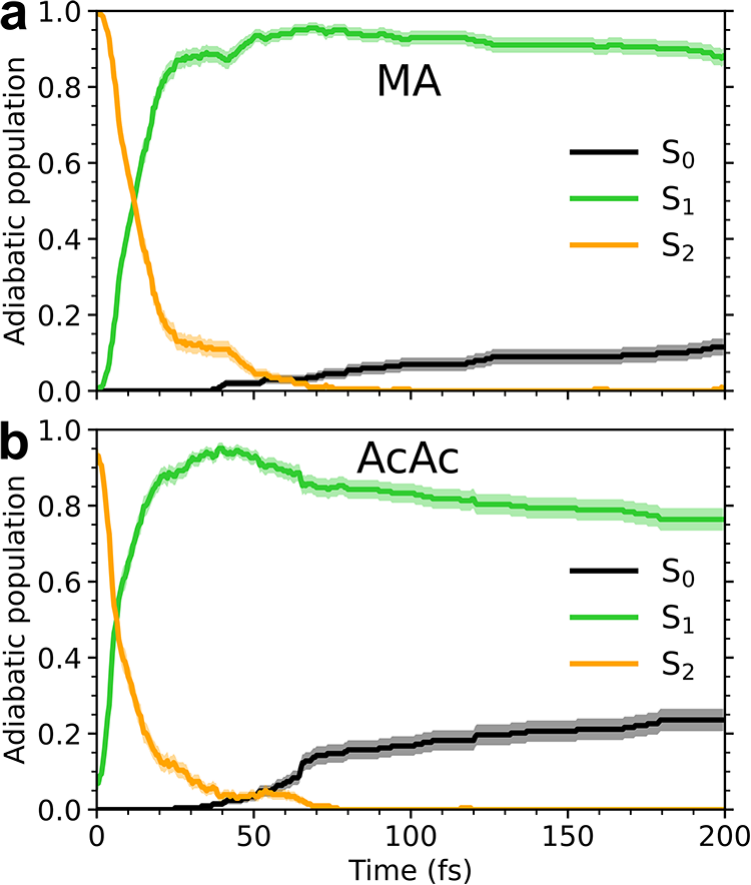
Adiabatic population dynamics following photoexcitation to the bright (ππ*) state in (a) MA and (b) AcAc. Shaded regions represent one bootstrap standard deviation obtained from 2000 samples. For the ICs, 199/200 for MA and 188/203 for AcAc had S_2_ as the bright state, while S_1_ was the bright state for the remaining ICs.

#### 3.2.1 Evolution of the Wavepacket toward the HTI Seam

To understand the structural motion mediating the ultrafast internal conversion, we examined the progress of the nuclear density along key coordinates. Figure 5 summarizes the results for BLA, HT, and SOA, while the projection onto the PyrC and torsional coordinates can be found in Figure S19. The line-outs (right plots) highlight the nuclear distribution in the FC region (black lines, as given by the IC distribution), on S_2_ (from the FC region until surface hop to S_1_, orange lines), and on S_1_ immediately following population transfer (defined by the behavior 5 fs after a surface hop, green lines). Furthermore, Figure 6 shows the distributions of geometric parameters reflecting the S_2_/S_1_- and S_1_/S_0_-nonadiabatic transfer events (i.e., hopping geometries).

**Figure 5:**
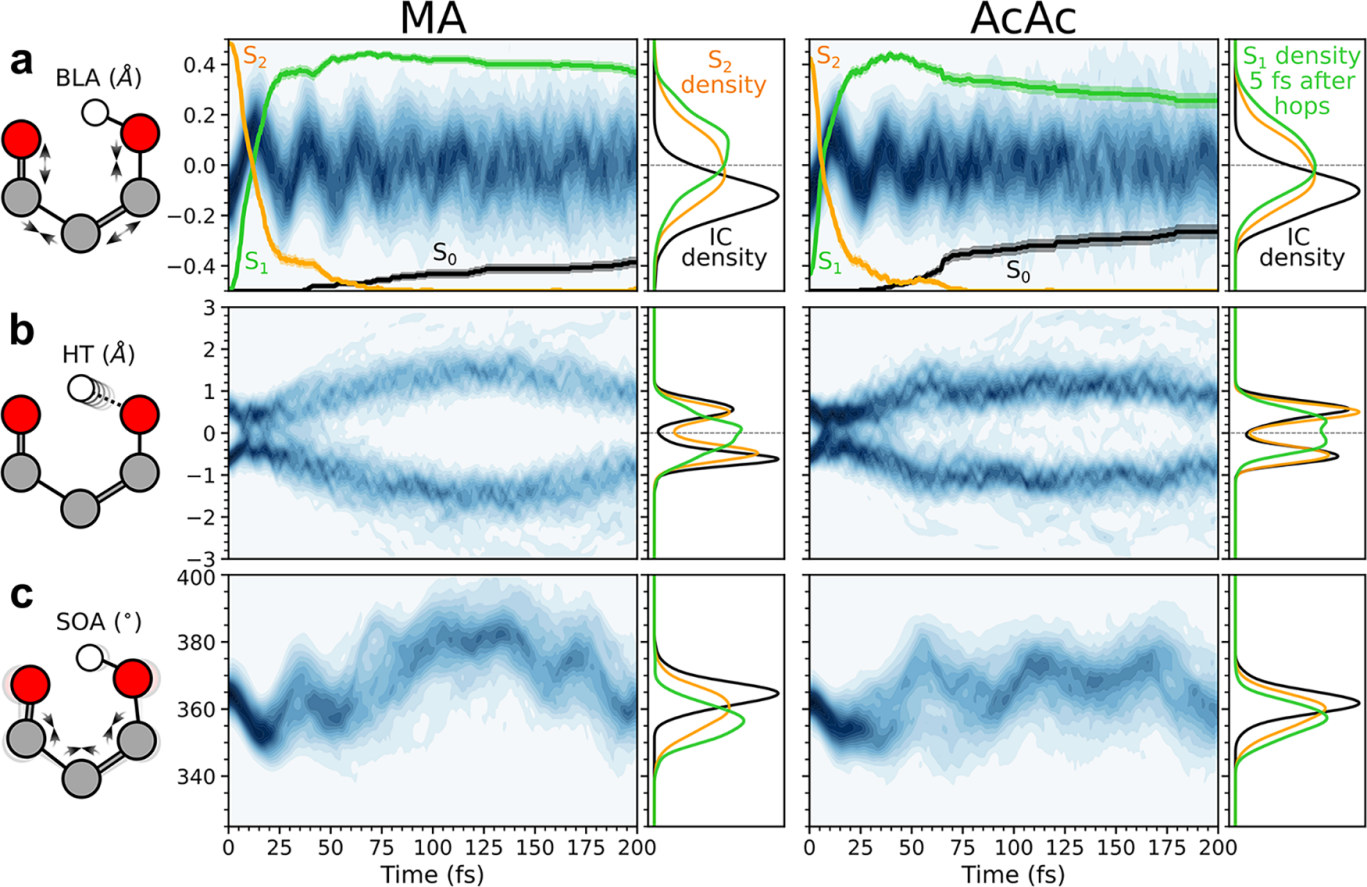
Time evolution of the nuclear density along the (a) BLA, (b) HT, and (c) SOA coordinates within 200 fs after photoexcitation for (left column) MA and (right column) AcAc. The adiabatic population traces from Figure 4 have been overlaid (no explicit *y*-axis but spans the range from 0 to 1) to highlight the change in population transfer (orange: S_2_; green: S_1_; black: S_0_). The one-dimensional distributions on the right show how the nuclear distribution changes from the FC region and up to 5 fs after population transfer to S_1_: black lines represent the IC distribution; orange lines represent the integrated S_2_ density; and green lines represent the S_1_-density integrated over the initial 5 fs immediately following the S_2_/S_1_-hop. The reduced nuclear densities were generated by Gaussian convolution along the respective geometric dimensions (standard deviations for BLA: 0.04 Å; HT: 0.1 Å; SOA: 2°).

**Figure 6:**
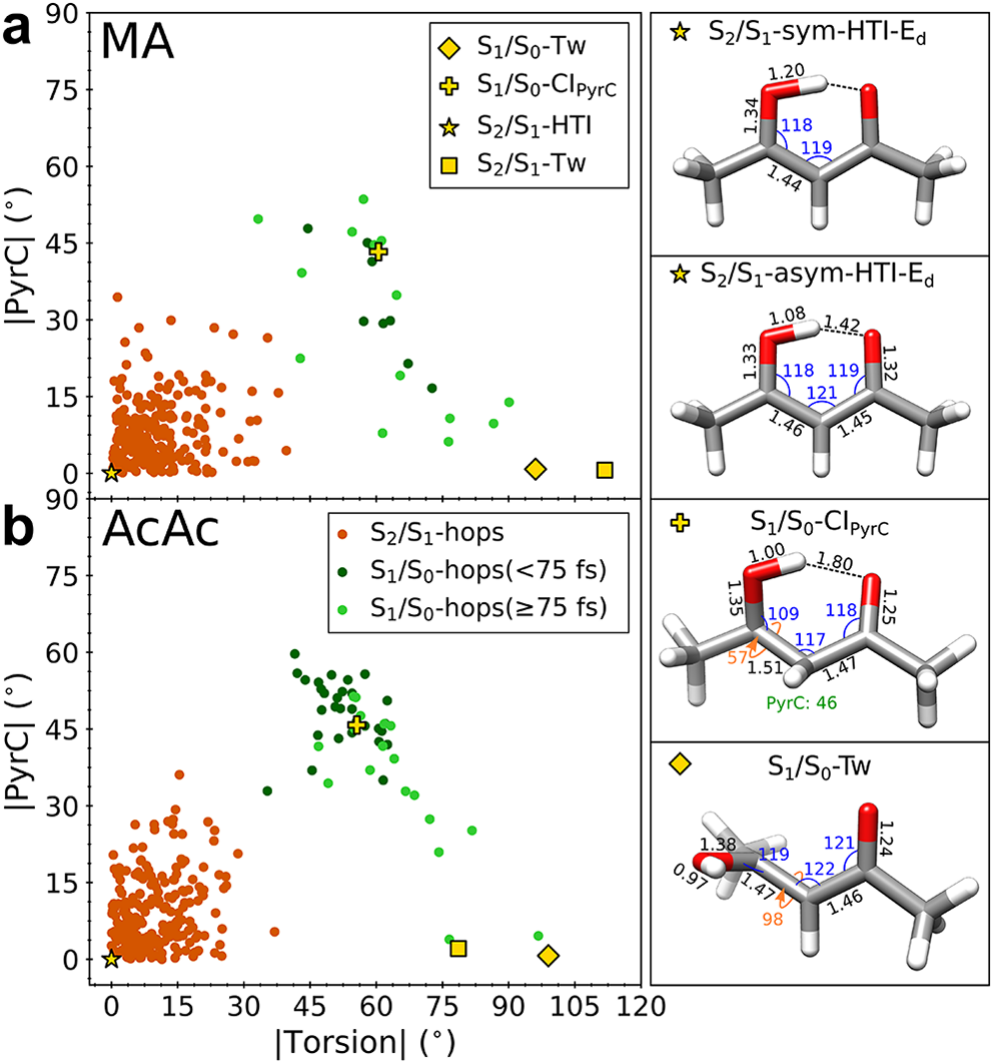
Differentiating the S_2_/S_1_-nonadiabatic transfer events (i.e., surface hops) from the early and late S_1_/S_0_ transfer events in the space spanned by PyrC and torsional modes for (a) MA and (b) AcAc. The S_2_/S_1_ transfer is mediated by in-plane motion near the HTI region, whereas the S_1_/S_0_ transfer is mediated by out-of-plane modes. In AcAc, the early S_1_/S_0_-hops clearly show a preference to be more centrally pyramidalized, with the late hops becoming increasingly twisted. Red, dark-green, and lime-green solid circles represent the S_2_/S_1_-hops, the early S_1_/S_0_-hops (<75 fs), and the late S_1_/S_0_-hops (≥75 fs), respectively. The golden markers represent the locations of the MECIs. The structures for the dynamically important symmetric and asymmetric S_2_/S_1_–HTI, S_1_/S_0_-Tw, and S_1_/S_0_-CI_pyrC_ intersection points for AcAc are shown on the right. Bond lengths in Å and (dihedral) angles are in degrees.

Upon photoexcitation to the ππ* state, the nuclear wavepacket proceeds coherently along the BLA coordinate for ∼2 periods (period time of ∼30 fs). The nuclear distribution on S_2_ shifts from the initially negative BLA value characterizing the FC region to BLA equalization. This brings the wavepacket toward the HTI seam and mediates the observed ultrafast population transfer to S_1_. The behavior along the HT coordinate shows that although the distribution slightly shifts toward reduced HT values on S_2_, it remains bimodal. As such, population transfer occurs predominantly near the asymmetric part of the HTI seam. As expected from the static picture, the asymmetry is somewhat reduced in MA. However, the inertia acquired on S_2_ along the BLA coordinate persists immediately after population transfer and leads to a transient near-unimodal S_1_ distribution along the H-transfer coordinate, which indicates motion of the hydrogen. The ultrafast dynamics is also characterized by a significant but slower contraction of the H-chelate ring (quantified by a ∼10° reduction of the SOA coordinate) as reminiscent of the progress toward the HTI seam. This early contraction is dominated by the ∠C­(C=O) keto (and less enol) bending mode (Figure S20) and its oscillatory motion persists on S_1_.

Until now, we have considered only in-plane modes. In fact, there is no substantial coherent out-of-plane motion during the initial 25 fs in any of the systems (see Figure S19). In particular, the twisted S_2_/S_1_-intersection seam is not accessed in the XMS-CASPT2 dynamics. By contrast, earlier CASSCF-based studies^42,44,45^ reported a ∼50 fs decay and a branching between the HTI (∼75–80%) and torsional (∼20–25%) pathways (ref 46 only reported C=O stretch-mediated transfer). This discrepancy between the branching ratios likely stems from two main factors: (i) use of different electronic-structure methods. In particular, the S_2_/S_1_-energy gap near the FC region is considerably smaller at the XMS-CASPT2 level (SA3-XMS­(Im=0.3)-CASPT2­(10,8)/cc-pVDZ (SSSR): 0.4 eV) than at the CASSCF level (SA3-CASSCF­(10,8)/cc-pVDZ: 1.2 eV). As discussed further in Section S2, this gap is highly method-dependent, with XMS-CASPT2 giving values at the lower end of the range, and (ii) differences in IC sampling methodology. Specifically, our simulations employ QT-AIMD sampling combined with energy-based filtering corresponding to a 266 nm pump pulse, whereas the CASSCF-based simulations sampled from a harmonic Wigner distribution of the vibrational ground state without applying selection criteria. The 266 nm pump lies on the red side of the absorption spectrum and therefore selects ICs with smaller S_2_/S_1_-energy gaps compared to sampling across the entire absorption band (see also Figure S11). Together, these factors may promote access to the HTI region and accelerate population transfer in our XMS-CASPT2 dynamics.

#### 3.2.2 S_1_-Trapping or Rapid S_0_-Recovery via Twist-Pyramidalized Seam

We now turn to the structural dynamics on S_1_ to better understand the early faster ground-state repopulation stage observed in AcAc—a feature that is absent for MA. Figure 6 compares the location of the S_2_/S_1_- and subsequent S_1_/S_0_-nonadiabatic transfer events along the C=C torsional and PyrC coordinates (Figure S21 shows the corresponding projections along the BLA/HT coordinates). Overall, the divisions between the two types of intersection seams are very similar for both molecules. The S_2_/S_1_-hopping geometries (red solid circles) cluster around planar geometries, consistent with the HTI seam (S_2_/S_1_–HTI-MECIs). On the other hand, the S_1_/S_0_-hops (green solid circles) involve substantial displacements along both the torsional and PyrC coordinates. As discussed above, the S_0_-repopulation in AcAc displays two types of growth: a ballistic rise occurring within 75 fs and a subsequent slower growth. Separating the S_1_/S_0_-hops by this 75 fs temporal threshold reveals that the early transfers (dark green) in AcAc are preferentially more pyramidalized, whereas the few later ones (light green) tend toward a larger degree of twisting. As expected from the lack of a fast S_0_-repopulation component, such a demarcation in the S_1_/S_0_-transfer events is largely missing in MA. We therefore turned to AcAc to uncover the structural basis of its ballistic ground-state recovery.

The faster component in AcAc arises from a smaller portion (∼15%) of the nuclear density that is initially guided toward a higher-lying, nonstationary part of the twisted S_1_/S_0_-intersection seam that features substantial pyramidalization of the central C atom while retaining the intramolecular H-bond. This part of the seam is also characterized by significant contraction of the H-chelate ring (SOA) mediated mostly by ∠C­(C=O). A MECI search starting from a representative hop geometry from the dynamics (and terminated early, upon locating the seam) suggests that this twist-pyramidalized region (denoted S_1_/S_0_-CI_PyrC_, Figure 6) of the seam is almost isoenergetic with the S_1_-minimum and thus located ∼0.5 eV above the twisted minimum on the S_1_/S_0_-intersection seam (S_1_/S_0_-Tw). Beyond this early decay, reaching the twisted S_1_/S_0_-intersection is a rarer event due to a torsional barrier (transition state located ∼0.25 eV (∼0.35 eV for MA) above the planar S_1_-minimum), where the S_1_ state gradually acquires ππ* character as shown in Figure 2. Accordingly, the majority of the population remains (transiently) trapped near the planar, ring-expanded S_1_-minimum throughout the duration of our simulations. As expected from the two distinct behaviors of AcAc, the electronic character of S_1_ along these pathways differs: the portion that undergoes ballistic ground-state recovery retains ππ* character upon reaching S_1_, whereas the trapped population near the S_1_-minimum gains nπ* character. These two behaviors are exemplified by two representative trajectories in Figure 7. Such differences in S_1_-valence character are expected to yield distinct fingerprints in oxygen *K*-edge X-ray absorption spectroscopy, providing a potential means for experimental discrimination.^108,113^

**Figure 7:**
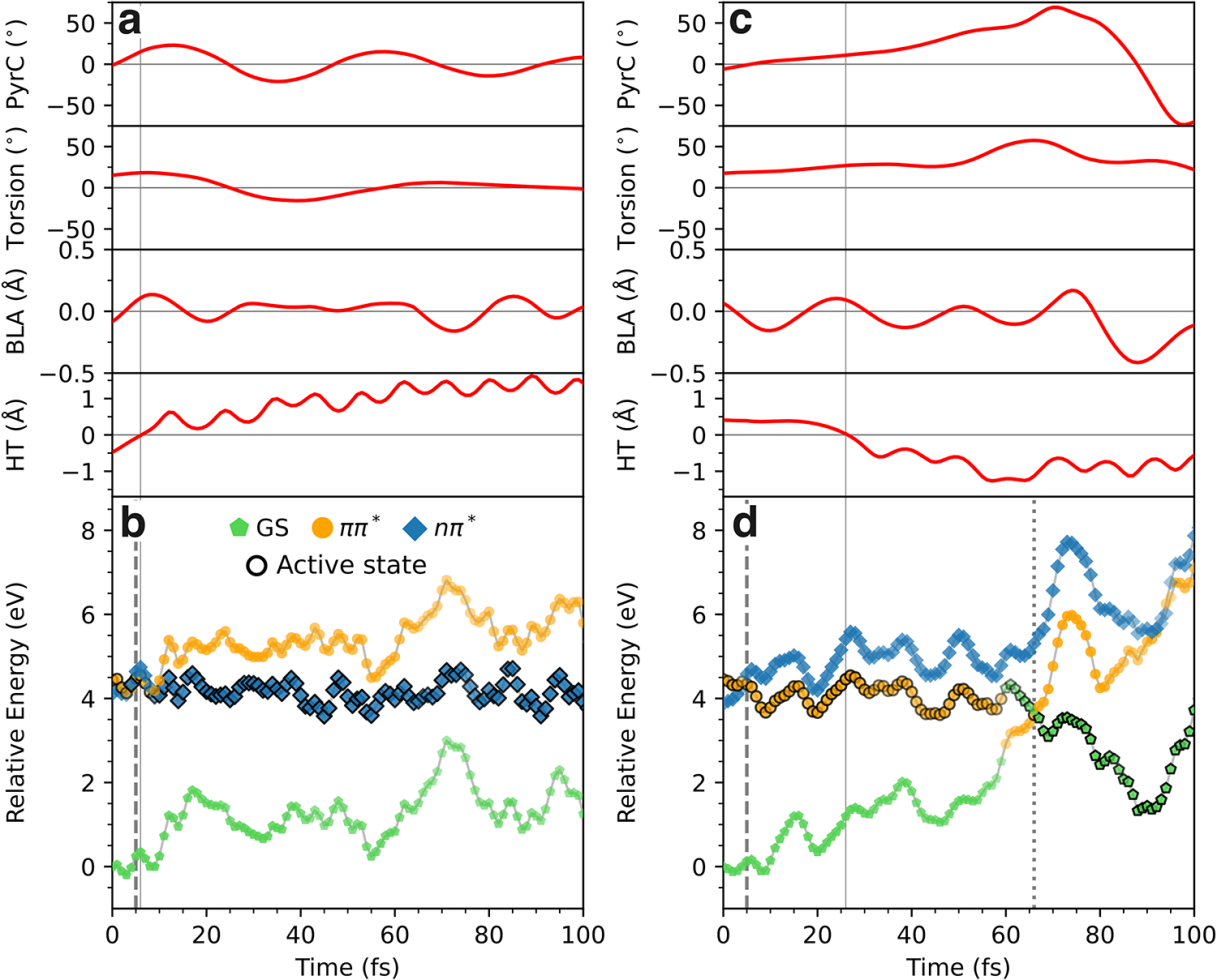
Electronic character of the singlet valence states in AcAc along two representative trajectories that proceed via (a, b) S_1_-trapping and (c, d) ballistic ground-state recovery. The colored markers show the leading electronic character. Time evolution of the key geometric parameters. Evolution of (a, c) key geometric parameters and (b, d) electronic character of the adiabatic states. The black outline on the markers indicates the active state. The transparency of the markers is proportional to the weight of the dominant contribution to the electronic wave function. Gray solid lines highlight the time point with HT = 0 Å, while the dashed and dotted lines indicate S_2_/S_1_- and S_1_/S_0_-hops, respectively.

A closer examination of the chelate-ring contraction (SOA in Figure 5c) reveals the origin of the differing ground-state repopulation behaviors in MA and AcAc. The methyl groups in AcAc have two key effects. First, they slow specific vibrational modes, particularly the keto ∠C­(C=O) bending mode. As a result, the full chelate-ring contraction is delayed, occurring at around 40 fs in AcAc, compared to 20 fs in MA. Accordingly, the contraction unfolds primarily on the S_1_ surface in AcAc, rather than proceeding more closely in sync with the BLA motion. Second, the added mass on the terminal carbon atoms introduces an inertial mismatch that specifically activates the methine hydrogen out-of-plane motion (PyrC). Together, these effects allow AcAc to access a higher-lying twist-pyramidalized part of the S_1_/S_0_-intersection seam, promoting early ground-state repopulation. In contrast, this pathway is basically unexplored in MA. Instead, the excess energy in MA is funneled into larger-amplitude in-plane ring breathing (i.e., SOA) motion around the S_1_-minimum (Figure 5c), which is generally more vigorous due to the fewer degrees of freedom. The slower structural envelope of the SOA coordinate is dominated by ∠CCC and ∠C­(C–O) bending, while the faster oscillations arise mainly from ∠C­(C=O) bending (Figure S20).

In summary, the valence dynamics of MA and AcAc display a shared initial response to S_2_(ππ*) photoexcitation, driven by BLA motion that steers both systems toward the HTI region of the S_2_/S_1_-intersection seam. In other words, the absence of methyl groups alone does not promote early C=C torsional motion at the XMS-CASPT2 level of theory. Rather, methyl-induced inertial effects shape subsequent ground-state recovery: AcAc exhibits both an early ballistic decay via a higher-lying twist-pyramidalized region of the S_1_/S_0_-intersection seam and a slower component resembling that of MA, where excess energy is primarily redirected into in-plane ring opening. These differences are evident by 200 fs, where the ground-state population of AcAc reaches ∼20–25% compared to ∼10% for MA.

This mechanistic picture contrasts with that of the smallest α,β-enones, acrolein, and its methylated derivatives, which share the C=C–C=O moiety.^10,12^ In enones,^31^ the absence of the enol group blue-shifts the S_2_(ππ*) state by ∼2 eV and red-shifts the S_1_(nπ*) state by ∼0.5 eV, enlarging the S_2_/S_1_-energy gap at the Franck–Condon point and leading to substantially higher excess kinetic energy once reaching S_1_. Dynamically, the S_2_/S_1_-deactivation occurs within ∼50 fs through a twist-pyramidalized part of the intersection seam.^12^ From there, ground-state recovery follows two comparably important pathways that resemble those in the enolones. Before significant planarization, part of the wavepacket accesses a nearby higher-lying twist-pyramidalized region of the S_1_/S_0_-intersection seam, leading to efficient, nearly concerted S_1_/S_0_-decay. The remainder relaxes toward the planar S_1_-minimum from which S_0_-repopulation occurs on a 0.9–3 ps time scale via torsional motion. Unlike in MA and AcAc, where a ∼0.3–0.4 eV barrier separates the S_1_-minimum and the lower-lying S_1_/S_0_-Tw MECI, the corresponding intersection seam in the enones is sloped and located 0.4–0.7 eV above the S_1_-minimum (with formyl methylation stabilizing the twisted S_1_/S_0_-MECI). Access to this seam is nonetheless facilitated by the high excess energy of the vibrationally hot S_1_ state, rendering this pathway an important ground-state recovery mechanism in enones.

Accordingly, the key mechanistic distinction between the enolones and enones following S_2_(ππ*) excitation lies in the departure route from the FC point. MA and AcAc decay through the HTI seam, whereas acrolein and its derivatives proceed via the twist-pyramidalized S_2_ pathway that enables a more direct and efficient return to the ground state through the geometrically proximal, higher-lying part of the S_1_/S_0_-intersection seam. In the enolones, the smaller excess kinetic energy is instead funneled predominantly into in-plane chelate-ring expansion, limiting access to the twisted S_1_/S_0_-intersection seam by intramolecular vibrational redistribution into the torsional mode.

#### 3.2.3 Comparison with Previous AcAc TRPES Studies

Our results for AcAc are broadly consistent with recent time-resolved photoelectron spectroscopy (TRPES). Squibb et al. observed a TRPES signal of the S_2_ state in AcAc but lacked the time resolution to determine its lifetime.^46^ Recently, Severino et al. confirmed this feature with sub-20 fs TRPES and directly resolved the ultrafast S_2_/S_1_-decay with a time constant of 23 ± 2 fs.^48^ In addition, they reported oscillations in the TRPES signal, which, based on accompanying XMS-CASPT2 dynamics (SA5/SA2 for singlets/triplets), were assigned to vibrational motion associated with deformations toward the ring-expanded S_1_-minimum, indicative of an ESIHT-mediated mechanism. Severino et al. also identified a distinct signature of the triplet state with a rise time of 1.6 ± 0.1 ps in line with a previous carbon *K*-edge TRXAS study (1.5 ± 0.2 ps).^53^ Their XMS-CASPT2 simulations predicted ∼45% internal conversion to S_0_, ∼39% population remaining near the S_1_-minimum, and ∼16% intersystem crossing by 700 fs. It should be noted that the TRPES signature of the triplet state becomes distinct only after relaxation toward the twisted *T*_1_-minimum, while ISC may occur earlier near the planar S_1_-minimum, where the S_1_, *T*_1_, and *T*_2_ states are nearly degenerate. Accordingly, the reported decay (∼1.5 ± 0.1 ps) of the TRPES signal associated with the S_1_-minimum likely represents an upper bound to the true S_1_-lifetime.

Three subtle differences nevertheless emerge when comparing our results with the XMS-CASPT2 simulations by Severino et al.^48^ First, they did not explicitly report the ballistic ground-state recovery component observed in our simulations, characterized by pyramidalization of the methine C atom and intermediate C=C torsion. Second, their theory level, employing state-averaging over five states, suggests a lower torsional barrier on S_1_ (∼0.18 eV, Table S6 in ref 48) than we obtain with three-state-averaging (∼0.26 eV). This difference might account for the less pronounced S_0_-repopulation found in our work (compared to ∼30% at 200 fs in the Severino work). Third, our simulations were restricted to the singlet manifold and therefore exclude ISC. The higher torsional barriers on S_1_ found in this work suggest a longer S_1_-lifetime and potentially a branching ratio shifted toward a higher triplet yield. Quantifying these aspects and their sensitivity to methodological details, including IC generation, electronic-structure level, and nonadiabatic dynamics method, will be an important target for future studies.

While TRPES has established the ultrafast time scale of the S_2_ state and revealed spectral remnants on S_1_ consistent with an ESIHT mechanism, it does not directly resolve the specific motion that mediates S_2_/S_1_-internal conversion. Below, we consider the extension to *K*-edge time-resolved oxygen X-ray photoelectron spectroscopy (TRXPS) as a means to probe the evolving local structure at the two oxygen atoms.

### 3.3 Time-Resolved X-ray Photoelectron Spectroscopic Fingerprints

The localized nature and chemical sensitivity of X-ray spectroscopic probes make them well-suited to tracking ultrafast dynamics at the two oxygen sites. While we have previously proposed TRXAS at the oxygen *K*-edge as an electronic probe of valence-state character and competing pathways in MA,^108^ we here explore the complementary potential of TRXPS. Specifically, we report oxygen *K*-edge XPS fingerprints along interpolated paths and representative pathways of the earliest decay in AcAc observed in our XMS-CASPT2 dynamics. This pathway-based approach highlights how TRXPS could serve as a probe of symmetric ESIHT in future experiments. Compared with TRXAS, TRXPS does not encode valence-orbital character. While this might seem like a limitation, it enhances its role as a structural probe: TRXAS intensities depend sensitively on the interaction-weighted overlap between the 1s-orbital and the singly occupied molecular orbital, whereas in TRXPS, the near-identical shapes of the core orbitals leave the pre-edge photoionization cross sections largely comparable (see discussion above). As a result, TRXPS emphasizes chemical shifts instead of valence-orbital character.

As a baseline, Figure 8 compares the experimental static XPS spectrum of AcAc to the simulated counterpart. The two pre-edge features can be explained by a chemical-shift argument within a C_*s*_ ground-state description: the ketonic O pre-edge feature is red-shifted by ∼1.4 eV relative to the enolic feature due to increased electronic screening. This pre-edge splitting is smaller by ∼1 eV than the pre-edge gap in the static XAS spectrum of AcAc (∼2.4 eV).^114^ An alternative interpretation proposed by Feyer et al. invokes H-tunneling in the *C*_2*v*_-ground state combined with keto- or enol-localized vibrational wave functions in the core-ionized states,^92^ underscoring that the correct ground-state vibrational description remains an open question. We will leave this aspect for future work and instead turn to transient oxygen *K*-edge XPS signatures along the two decay pathways.

**Figure 8:**
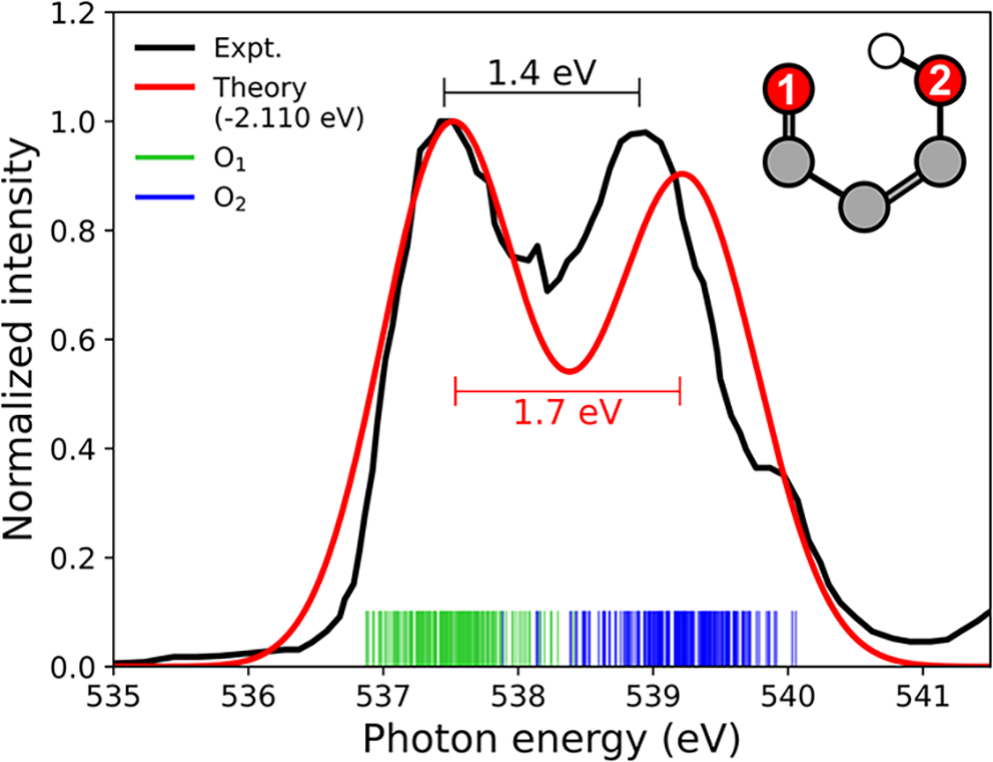
Comparison of the Experimental and Computed Static XPS Spectra of AcAc. Ionization energies were computed for each initial condition, and the spectra were convolved with a Gaussian line shape of full-width-half-maximum of 1.0 eV, broader than the O 1s core-hole lifetime due to the limited sampling. We assumed unity cross sections for all transitions. Only the two pre-edge features from each oxygen were included in the simulated XPS spectrum, which was uniformly shifted by −2.110 eV to align with the first experimental pre-edge peak. The ionization energies were obtained at the RASPT2 level of theory. The experimental gas-phase spectrum was digitized from ref 92 using WebPlotDigitizer.^115^ The experimental pre-edge maxima are located at 537.5 and 538.9 eV, while the shoulder at ∼540 eV was assigned to residual water in the vacuum.

Figure 9 shows the transient oxygen *K*-edge signatures associated with the dynamics, represented in two complementary ways: (a,b) simplified interpolated pathways and (c,d) representative trajectories from Figure 7. H-transfer is marked by coalescence of the two oxygen pre-edge features, reflecting the near-equivalence of the O atoms at approximate *C*_2*v*_-symmetric geometries. By contrast, twist-pyramidalization produces a substantial blue-shift (∼2–3 eV) of both pre-edge features, reflecting the reduced O 1s core-hole screening that accompanies the breaking of the π-conjugation. As is evident from the representative trajectories, this merging is expected irrespective of which valence state is populated. In TRXAS, the signals are convolved with large intensity differences: on S_2_, the two pre-edge features are of comparable intensity (keto:enol ratio of 1:2–4), whereas on S_1_, the keto pre-edge is about an order of magnitude stronger than the enol (keto:enol ratio ∼10:1).^108^ Consequently, the weaker feature becomes masked, and the spectrum effectively appears as a single peak. In contrast, the comparable pre-edge intensities in TRXPS render the coalescence during H-transfer discernible.

**Figure 9:**
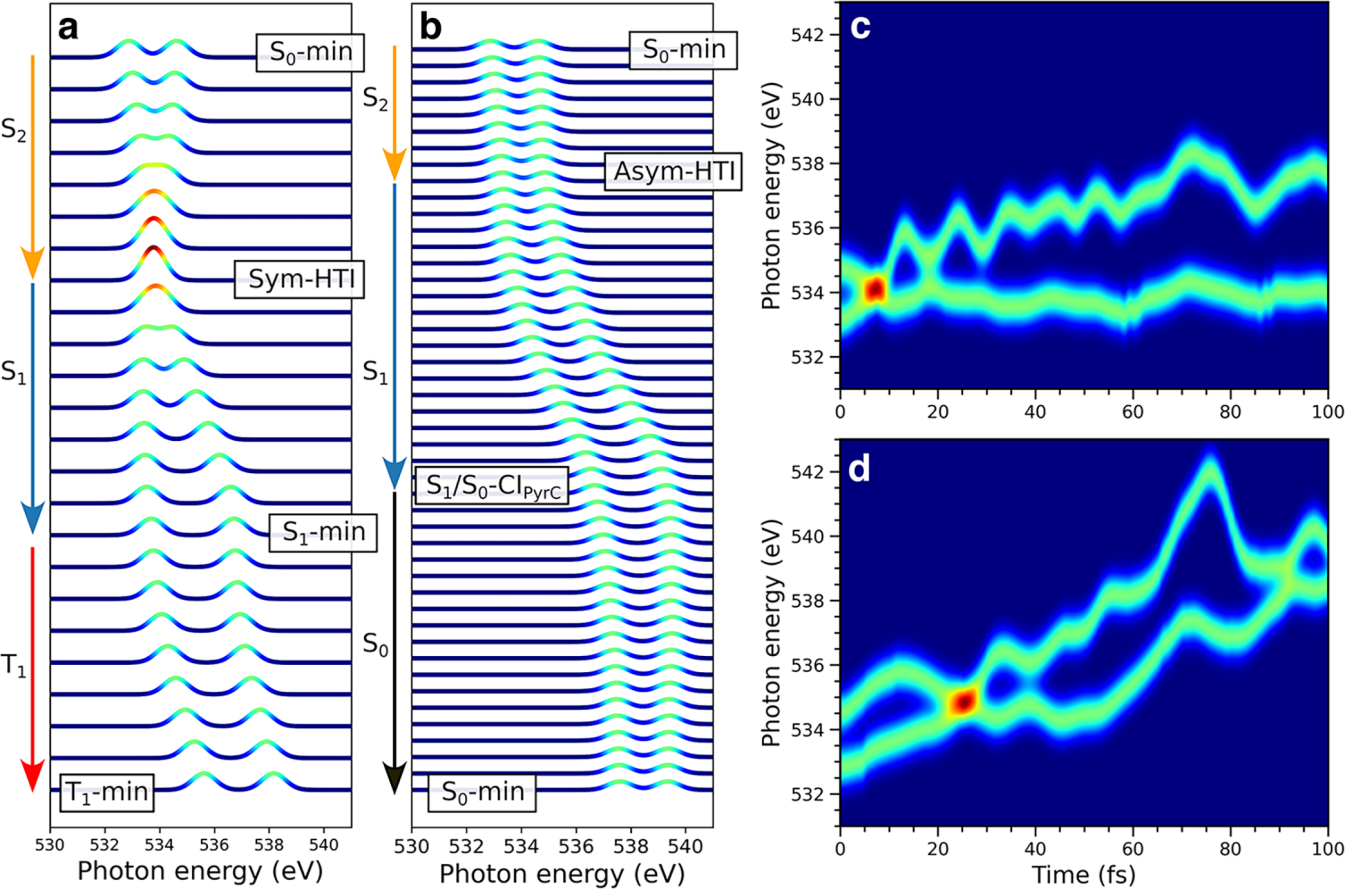
Simulated oxygen *K*-edge ionization energies along the decay pathways of AcAc. Picture emerging along geodesic interpolated pathways: (a) H-transfer mediated pathway with signatures of intersystem crossing to T_1_ (ESIHT pathway in Figure 2); and (b) S_2_/S_1_-internal conversion through the asymmetric HTI seam followed by ballistic decay to the ground state through a nonstationary twist-pyramidalized region of the S_1_/S_0_-intersection seam. This path is an idealized representation of the dynamically accessed ballistic ground-state recovery pathway rather than the torsional pathway in Figure 2. The valence electronic state is indicated by colored arrows, and geometries for the end points of each interpolated segment are highlighted in the boxes. The TRXPS map along the two representative trajectories in Figure 7 and is analogous to the interpolated paths: (c) decay near the symmetric part of the HTI seam and trapping on S_1_. H-transfer occurs at ∼6 fs, and (d) ballistic ground-state recovery with H-transfer occurring at ∼26 fs. Stick spectra for the two pre-edge features were obtained at the RASPT2 level, convolved with a Gaussian line shape of fwhm 1.0 eV and uniformly shifted by −2.110 eV.

Together, TRXPS and TRXAS therefore provide complementary windows into the dynamics: TRXPS can serve as a direct structural probe of the H-transfer, while TRXAS, through its valence-orbital sensitivity, could allow for probing the lifetime of the S_1_(nπ*) state.

## 4 Conclusion and Outlook

We have investigated the earliest ultrafast decay in the enolone HO–C=C–C=O motif with XMS-CASPT2-based nonadiabatic dynamics simulations, focusing on how methylation modifies the potential-energy landscape and the ensuing dynamics by comparing MA and AcAc. While methyl groups are often thought of as inertial substituents, their weakly electron-donating character also modulates PESs of charge-polarized regions of configurational space, such as relative energetics of nπ* and ππ* states.

Our simulations show that both molecules exclusively undergo H-transfer-mediated S_2_/S_1_-internal conversion driven by BLA that guides the nuclear wavepacket to the HTI seam on a sub-20 fs time scale. The population decay occurs predominantly near the asymmetric parts of the HTI seam, while actual H-motion takes place mainly on S_1_, followed by chelate-ring contraction and subsequent expansion. While methylation leaves the fundamental S_2_/S_1_-decay pathway unchanged, it subtly reshapes the ensuing motion on S_1_. Its weak electronic effects extend the asymmetry of the HTI seam, and the added mass slows the chelate-ring contraction and introduces dynamical asymmetry between the central C–H and terminal C–CH_3_ groups. This imbalance promotes early out-of-plane motion, facilitating ballistic access to a higher-lying twist-pyramidalized region of the S_1_/S_0_-intersection seam within ∼100 fs. Consequently, AcAc exhibits both this early ground-state recovery followed by a slower torsional component similar to that in MA, resulting in somewhat different S_0_-populations after 200 fs (∼20–25% for AcAc and ∼10% for MA).

In comparison, α,β-enones, sharing the C=C–C=O motif, relax directly via a twist-pyramidalized pathway, which provides an efficient and almost concerted route to the ground state.^10–12^ In the enolones, the intramolecular H-bond introduces and redirects the wavepacket through the HTI seam and instead transiently traps a larger part of the population near the planar S_1_-minimum. Excess energy is mainly dissipated into the in-plane modes rather than driving torsion-mediated S_1_/S_0_-internal conversion. This mechanistic difference explains the markedly lower ground-state recovery in MA and AcAc compared with the enones.

While our considerations were restricted to the singlet manifold, the proximity of the T_1_(ππ*) state near the S_1_(nπ*)-minimum indicates that ISC should play a role.^107^ This picture is consistent with a recent TRPES study of AcAc, which resolved the ultrashort lifetime of the S_2_ state, vibrational remnants on S_1_ induced by the S_2_/S_1_-decay, and the emergence of a triplet fingerprint on the ps-time scale.^48^ In this work, we identified oxygen *K*-edge TRXPS as a potential experimental route to directly track the ultrafast BLA and H-motion governing S_2_/S_1_-decay by leveraging its sensitivity to the local chemical shifts at the enolic and ketonic O atoms. Remaining open questions concern the S_1_-lifetime and the ps-time scale branching ratio between triplet formation and ground-state recovery. The corresponding oxygen *K*-edge TRXAS observable allows monitoring nπ* populations through their intense pre-edge features,^108,113^ and hence holds potential to determine the S_1_-lifetime. We hope that our theoretical work will inspire further experiments combining complementary modalities to fully map the ultrafast dynamics in these prototype systems.

## Supplementary Material



## Data Availability

10.5281/zenodo.17210367: xyz files for critical points at the XMS-CASPT2 levels of theory. Quantum-thermostat generated initial conditions (positions and momenta) used in the FSSH simulations.

## Notes

Electronic-structure theory validation, critical point analysis, definitions of geometric parameters, details on initial condition sampling, and additional dynamics analyses (PDF)

## §

School of Chemistry, Pharmacy and Pharmacology, University of East Anglia, Norwich NR4 7TJ, U.K
