# Supplementary material for: Electronic and Inertial Effects of Methylation on Excited-State Hydrogen Transfer
Source: J Phys Chem A. 2026 Jan 27;130(5):1090–103. doi: 10.1021/acs.jpca.5c07439 (PMC12884517; doi:10.1021/acs.jpca.5c07439)
Supplement: Supplementary file 1 [file jp5c07439_si_001.pdf]

Supporting Information: Electronic and Inertial Effects of Methylation on Excited-State Hydrogen Transfer

Pratip Chakraborty,[†,¶] Rafael C. Couto,[†] and Nanna H. List*,[†,‡]

†*Department of Chemistry, KTH Royal Institute of Technology, SE 10044, Stockholm, Sweden*

‡*School of Chemistry, University of Birmingham, Birmingham B15 2TT, United Kingdom*

¶*Present address: School of Chemistry, Pharmacy and Pharmacology, University of East Anglia, Norwich NR4 7TJ, United Kingdom*

E-mail: nalist@kth.se;n.h.list@bham.ac.uk

Contents

S1 Atom numbering and geometric parameter definitions **S3**

S2 Validation of electronic-structure level **S6**

S3 Active-space stability and trajectory monitoring **S15**

S4 Initial conditions for excited-state dynamics **S21**

S4.1 Sampling approaches S21

S4.2 Challenges in IC-sampling of AcAc S22

S4.3 Absorption spectrum S23

S4.4 QT-AIMD vs. harmonic Wigner sampling S23

S5 Additional analyses of the dynamics **S25**

References **S36**

S1 Atom numbering and geometric parameter definitions

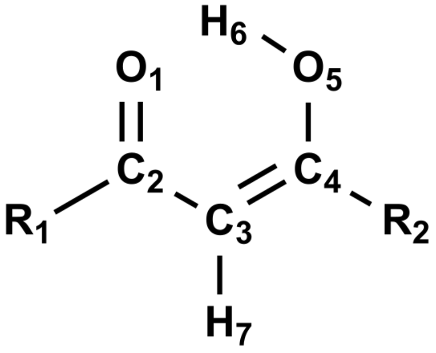

Figure S1: Atom numbering for malonaldehyde (MA: $R_{1,2}$=H) and acetylacetone (AcAc: $R_{1,2}$=CH_3).

Definitions of key distance parameters

$$\mathrm{HT} = (h_1 - h_2); \qquad h_1 = r_{\mathrm{O}_5-\mathrm{H}_6}; \qquad h_2 = r_{\mathrm{O}_1-\mathrm{H}_6} \tag{S1}$$

$$\mathrm{BLA} = \begin{cases} r_{\mathrm{O}_1-\mathrm{C}_2} - r_{\mathrm{C}_2-\mathrm{C}_3} + r_{\mathrm{C}_3-\mathrm{C}_4} - r_{\mathrm{C}_4-\mathrm{O}_5} & \text{if } h_1 < h_2 \text{ at IC} \\ -r_{\mathrm{O}_1-\mathrm{C}_2} + r_{\mathrm{C}_2-\mathrm{C}_3} - r_{\mathrm{C}_3-\mathrm{C}_4} + r_{\mathrm{C}_4-\mathrm{O}_5} & \text{if } h_1 \geq h_2 \text{ at IC} \end{cases} \tag{S2}$$

and angle-related parameters

$$\mathrm{SOA} = \angle\mathrm{O}_1\mathrm{C}_2\mathrm{C}_3 + \angle\mathrm{C}_2\mathrm{C}_3\mathrm{C}_4 + \angle\mathrm{C}_3\mathrm{C}_4\mathrm{O}_5 \tag{S3}$$

$$\mathrm{Torsion} = \begin{cases} \text{Dihedral } \angle\mathrm{C}_2\mathrm{C}_3\mathrm{C}_4\mathrm{O}_5 & \text{if } h_1 < h_2 \text{ at final } \mathrm{S}_1 \text{ time-step} \\ \text{Dihedral } \angle\mathrm{O}_1\mathrm{C}_2\mathrm{C}_3\mathrm{C}_4 & \text{if } h_1 \geq h_2 \text{ at final } \mathrm{S}_1 \text{ time-step} \end{cases} \tag{S4}$$

$$\text{O-H dihedral} = \begin{cases} \text{Dihedral } \angle\mathrm{C}_3\mathrm{C}_4\mathrm{O}_5\mathrm{H}_6 & \text{if } h_1 < h_2 \text{ at final } \mathrm{S}_1 \text{ time-step} \\ \text{Dihedral } \angle\mathrm{H}_6\mathrm{O}_1\mathrm{C}_2\mathrm{C}_3 & \text{if } h_1 \geq h_2 \text{ at final } \mathrm{S}_1 \text{ time-step} \end{cases} \tag{S5}$$

$$\mathrm{PyrC}_3 = \arccos((\mathbf{e}_{\mathrm{C}_2\text{-}\mathrm{C}_3} \times \mathbf{e}_{\mathrm{H}_7\text{-}\mathrm{C}_3}) \cdot \mathbf{e}_{\mathrm{C}_4\text{-}\mathrm{C}_3}) - \frac{\pi}{2} = \mathrm{PyrC} \tag{S6}$$

$$\mathrm{PyrC}_2 = \arccos((\mathbf{e}_{\mathrm{O}_1\text{-}\mathrm{C}_2} \times \mathbf{e}_{\mathrm{R}_1\text{-}\mathrm{C}_2}) \cdot \mathbf{e}_{\mathrm{C}_3\text{-}\mathrm{C}_2}) - \frac{\pi}{2} \tag{S7}$$

$$\mathrm{PyrC}_4 = \arccos((\mathbf{e}_{\mathrm{C}_3\text{-}\mathrm{C}_4} \times \mathbf{e}_{\mathrm{R}_2\text{-}\mathrm{C}_4}) \cdot \mathbf{e}_{\mathrm{O}_5\text{-}\mathrm{C}_4}) - \frac{\pi}{2} \tag{S8}$$

Here, $\mathrm{e}_{\mathrm{Y-X}}$ denotes a unit vector pointing along the bond from atom X to atom Y. The pyramidalization angle PyrC_3 represents the central C pyramidalization (referred to as PyrC in the main text), whereas PyrC_2 and PyrC_4 are connected to either a H-atom (in MA) or a methyl group (AcAc). With this definition of pyramidalization, an idealized sp^2 C-atom would give 0° while an idealized sp^3 C-atom, as in methane, corresponds to 55°.

Table S1: Computational details for the decoherence-corrected FSSH simulations and observable calculations of MA and AcAc.

	**Malonaldehyde**	**Acetylacetone**
**1. Initial condition (IC) generation and selection**		
Method	QT-AIMD	QT-AIMD
Program	ABIN/TeraChem	ABIN/TeraChem
Program version	1.1/1.9-dev-c07804d	1.1/1.9-dev-c07804d
Temperature (K)	298.15	298.15
QT parameters	GLE4MD (Ns=6, $\hbar\omega_{\max}/k_BT$=20)	GLE4MD (Ns=6, $\hbar\omega_{\max}/k_BT$=20)
ES method	B3LYP(D3-BJ)	B3LYP(D3-BJ)
Basis set	6-31G(d,p)	6-31G(d,p)
IC selection	Energy window	Energy window
Selection window (eV)	4.661±0.05	4.661±0.05
ES method	same as in 3	same as in 3
Spectral shift (eV)	+0.096	+0.096
**2. Nonadiabatic dynamics**		
Method	FSSH	FSSH
Program	SHARC	SHARC
Program version	3.0	3.0
Propagation time (fs)	200	200
#ICs	256	265
#runs per IC	1	1
#failed trajectories‡	(S_2: 5, S_1: 43, S_0: 8)	(S_2: 1, S_1: 58, S_0: 3)
Nuclear time step (fs)	0.5	0.5
Electronic time step (fs)	0.02	0.02
Momentum rescaling	NACV	NACV
Decoherence correction	Energy	Energy
Decoherence parameter (a.u.)	0.1	0.1
Reflection of frustrated hops	None	None
**3. Electronic-structure (ES) method**		
ES method	XMS-CASPT2	XMS-CASPT2
Program	BAGEL	BAGEL
Program version	1.2.2	1.2.2
Basis set	cc-pVDZ	cc-pVDZ
CAS (e,o)	10,8	10,8
IPEA shift (a.u.)	0.0	0.0
Imag shift (a.u.)	0.3	0.3
Frozen core	yes	yes
Contraction	SSSR	SSSR
State-averaging*	3S	3S
**4. Observable calculations**		
Observable	-	XPS
ES method	-	XMS-RASPT2
Program	-	OpenMolcas
Program version	-	24.06
Basis set	-	cc-pVDZ
$(n, l, m; i, j, k)$†	-	(11, 1, 0; 1, 12,0)
IPEA shift (a.u.)	-	0.0
Imag shift (a.u.)	-	0.3
Frozen core	-	no
State-averaging*	-	5D (for each O 1s)
Comments	-	photoionization cross-sections assumed unity

‡ See Table S6 for the trajectory monitoring and acceptance criteria employed.
* S: singlets; D: doublets; T: triplets.
† n is the number of active electrons, l the maximum number of holes in RAS1, m the maximum number of electrons allowed in RAS3, while i, j, k are the number of active orbitals in RAS1, RAS2, and RAS3, respectively.

S2 Validation of electronic-structure level

To assess if the more cost-effective SA3(singlets)/SA2(triplets)-XMS(Im=0.3)-CASPT2(10,8)/cc-pVDZ (SSSR) level of theory would be suitable for nonadiabatic dynamics simulations, we compared its critical point energies and geometries (minima and minimum energy conical intersections) with those obtained using the higher-level SA3/SA2-XMS(Re=0.3)-CASPT2(14,12)/cc-pVDZ (MSMR) reference. The two active spaces are displayed in Figure S2, and a discussion of the stability of the smaller active space during dynamics is provided in Section S3. The energy-level diagrams for the two levels of theory are shown in Figure S3. Tabulated energies and key geometric parameters are reported in Tables S4 and S5 for MA, with the AcAc counterparts summarized in Tables S2 and S3. In addition, benchmark calculations comparing the cc-pVDZ and aug-cc-pVDZ basis sets along the H-transfer coordinate in MA show negligible differences in relative singlet-state energetics (see Figure S4), indicating that diffuse functions do not play a significant role and motivating the use of the cc-pVDZ basis set.

To describe the conformational variations along the HTI seam, we adopt a naming convention based on: (i) whether the methyl groups are staggered (S) or eclipsed (E), where S designates the staggered conformer in which the keto-side methyl group has one of its C–H bonds oriented in the same direction as the adjacent C=O bond, (ii) for the eclipsed conformer, whether a C–H bond of each methyl group points in the same direction as the C–O bonds (u, up) or in the opposite direction (d, down), and (iii) whether the enolic H-atom is positioned symmetrically (sym) or asymmetrically (asym) relative to the O-atoms. Figure S5 shows the AcAc critical point geometries at the XMS-CASPT2(14,12) level, which serve to illustrate the adopted nomenclature: S_0-min corresponds to the S-conformer (see also Section S4), while S_1-min corresponds to the E_d-conformer.

Relative to the XMS-CASPT2(14,12) reference, the mean absolute errors (MAEs) of XMS-CASPT2(10,8) are 0.06 eV (MA) and 0.07 eV (AcAc) for singlet energies, and 0.08 eV (MA) and 0.09 eV (AcAc) for triplet energies, across all considered stationary points (i.e. ex-

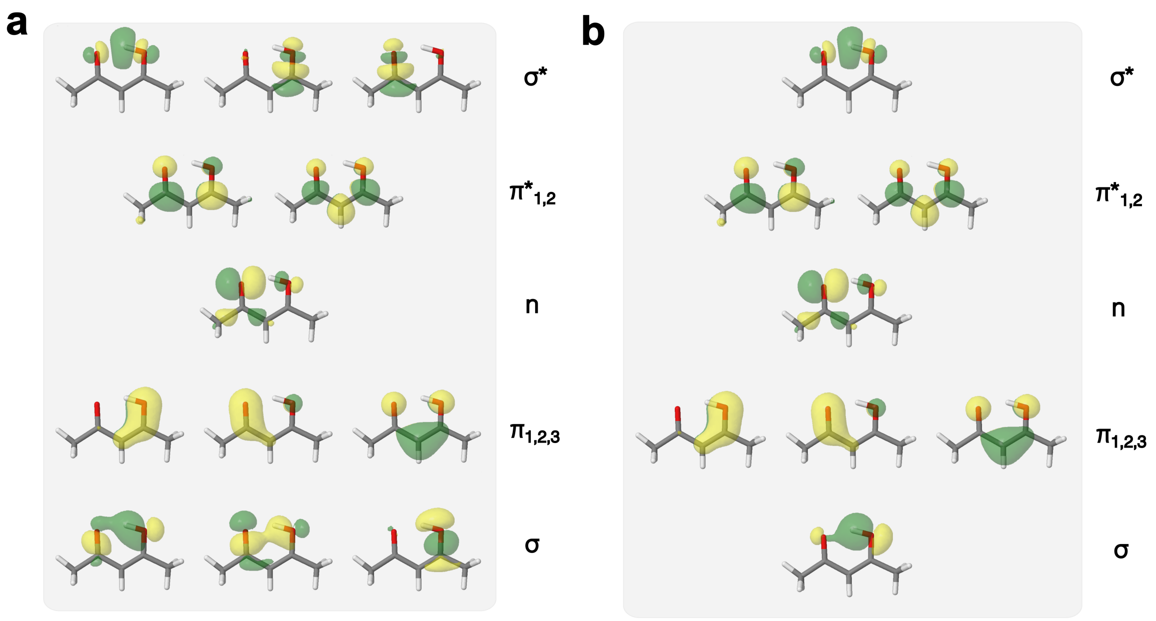

Figure S2: The active spaces employed in this study, shown for the staggered S_0-minimum of AcAc: (a) CAS(14,12) used for benchmark reference; and (b) CAS(10,8) used for optimizations and nonadiabatic dynamics simulations. Isovalue = 0.05 a.u.

cluding S_1/S_0-CI_{PyrC}). For MA, the above MAEs exclude the T_1-min, which is discussed separately below. Overall, the smaller active space reproduces the relative energetic ordering of the critical points but shifts energies by approximately +0.1 eV. For MA, the larger active space yields an S_1-min that is slightly non-planar and a T_1-min that is only weakly C=C twisted, whereas the smaller active space gives a planar S_1-min and a more strongly twisted T_1-min, both of which are consistently obtained for AcAc across both active spaces. The comparatively flatter T_1 potential energy surface likely contributes to the method sensitivity observed for this minimum. A similar sensitivity is reflected in the energetic ordering of the S_1, T_1 and T_2 states at the S_1-min geometry. With the larger active space, the two triplet states are essentially degenerate and lie slightly below S_1 (by ∼0.06 eV), whereas with the smaller active space, T_2 is ∼0.1 eV above both S_1 and T_1. Such discrepancies are expected for states that are energetically close in a region of configuration space (here, around S_1-min) but differ in their geometric sensitivity.

Spectroscopic measurements place the S_2 state about 4.7 eV above S_0 at the FC point

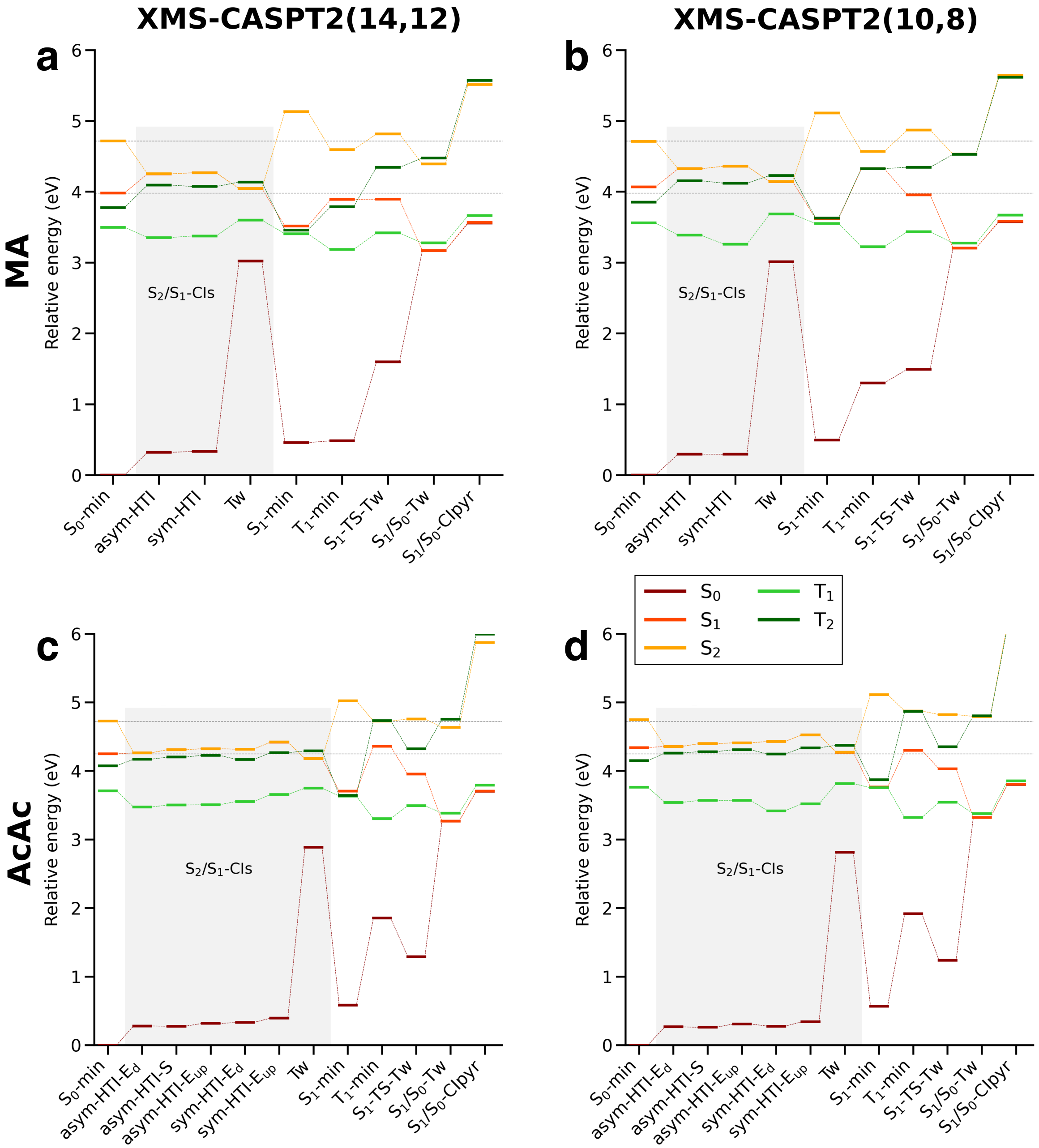

Figure S3: Comparison of critical point energies obtained at the XMS(Re=0.3)-CASPT2(14,12) and XMS(Im=0.3)-CASPT2(10,8) levels of theory for (a-b) MA and (c-d) AcAc relative to their respective S_0-min. The gray area highlights S_2/S_1-MECIs. The horizontal dashed lines correspond to the relative S_2- and S_1-energies at the S_0-min with the larger active space. The underlying energies are summarized in Tables S2 to S5.

for both MA and AcAc.[1–3] The S_2/S_1-gap is estimated to be <1.2 eV in MA,[1,4,5] while for AcAc it has been reported to be ∼0.7 eV (S_1 4.04 eV; S_2: 4.7 eV) with electron energy loss spectroscopy (EELS)[2] and 0.5 eV (S_1 4.2 eV; S_2: 4.72 eV) with UV-absorption.[3] For the

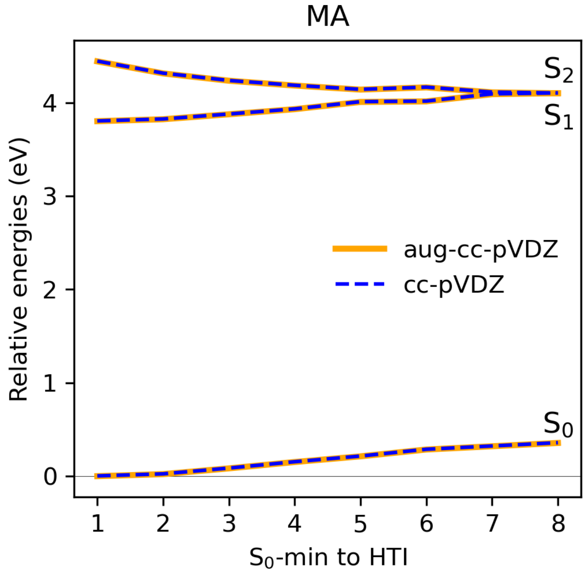

Figure S4: Effect of diffuse functions on H-transfer in MA. Comparison of the three lowest singlet-state energies along a geodesic interpolated path connecting the S_0-minimum and the HTI. The end points were optimized at the XMS(Re=0.3)-CASPT2(14,12) level using either the cc-pVDZ (blue, dashed) or the aug-cc-pVDZ (orange, solid) basis set, and the energies at all points along the path were evaluated at the corresponding level of theory.

SA3-XMS(Re=0.3)-CASPT2(14,12)/cc-pVDZ (MSMR) reference, we obtain an S_2 transition energy close to the experimental value of 4.7 eV and an S_2/S_1-energy gap consistent with the UV-absorption measurement. The cheaper SA3-XMS(Im=0.3)-CASPT2(10,8)/cc-pVDZ (SSSR) level retains the S_2 transition energy but reduces the S_2/S_1-gap by $\sim$0.1 eV. Taken together, these comparisons indicate that XMS-CASPT2(10,8) provides a robust description of the key energetic and structural features required for reliable dynamics.

Earlier CASSCF studies with different active spaces systematically overestimated excitation energies by more than 1 eV, particularly for the $S_2(\pi\pi^*)$ state, leading to artificially large S_2/S_1-energy gaps.[5,6] More recent works with correlated methods, including CC2,[7] EOM-CCSD,[8,9] ADC(2),[9] and TDDFT,[9] as well as (X)MS-CASPT2,[7,10–13] provide significantly improved relative energies, although they do not converge toward a single experimental reference, instead alternating between closer agreement with the EELS or UV-absorption measurement.

Table S2: SA3(and SA2)-XMS(Re=0.3)-CASPT2(14,12)/cc-pVDZ (MSMR) energies (eV) and important geometrical parameters for critical points optimized at the aforementioned level for AcAc. Energies are reported relative to the ground state energy at the FC point. MECIs are not confirmed to be minima on the intersection seam.

State/geom	FC (S)	S_1-min (E_d)	S_1-TS-Tw	T_1-min	asym-HTI-E_d	asym-HTI-S	asym-HTI-E_{up}	sym-HTI-E_d	sym-HTI-E_{up}	S_2/S_1-Tw	S_1/S_0-Tw	S_1/S_0-CI_{PyrC}
S_0	0.000	0.584	1.290	1.855	0.280	0.276	0.317	0.330	0.395	2.887	3.268	3.703
S_1	4.250	3.704	3.954	4.358	4.263	4.311	4.323	4.317	4.421	4.179	3.269	3.706
S_2	4.726	5.021	4.758	4.727	4.264	4.311	4.323	4.318	4.421	4.180	4.634	5.873
T_1	3.711	3.633	3.492	3.304	3.473	3.503	3.507	3.554	3.656	3.748	3.385	3.792
T_2	4.074	3.644	4.322	4.734	4.170	4.202	4.228	4.168	4.266	4.292	4.754	5.995
h_1 (Å)	1.006	0.972	0.998	0.980	1.081	1.075	1.058	1.202	1.197	0.971	0.971	0.997
h_2 (Å)	1.630	1.958	1.700	1.982	1.422	1.431	1.472	1.204	1.196	3.191	3.350	1.804
HT (Å)	-0.624	-0.986	-0.702	-1.002	-0.341	-0.356	-0.414	-0.002	0.001	-2.220	-2.379	-0.807
O_1-C_2 (Å)	1.251	1.351	1.302	1.249	1.317	1.320	1.317	1.339	1.345	1.309	1.240	1.249
C_4-O_5 (Å)	1.332	1.372	1.352	1.376	1.329	1.330	1.333	1.339	1.345	1.385	1.381	1.354
C_2-C_3 (Å)	1.458	1.404	1.420	1.450	1.445	1.446	1.441	1.435	1.429	1.380	1.461	1.472
C_3-C_4 (Å)	1.381	1.402	1.458	1.497	1.463	1.458	1.465	1.435	1.429	1.467	1.470	1.506
O_1-O_5 (Å)	2.560	2.777	2.568	2.802	2.462	2.464	2.484	2.369	2.345	3.545	3.608	2.685
$PyrC_3$ (°)	0.0	0.0	-7.4	7.6	0.0	0.0	0.0	0.0	0.0	1.5	-0.2	46.1
$\angle C_2C_3C_4$(°)	120.0	126.5	120.6	120.1	120.6	120.7	120.9	119.1	119.0	125.6	121.6	117.4
$\angle O_1C_2C_3C_4$(°)	0.0	0.0	4.1	-8.6	0.0	0.2	0.1	-0.2	0.0	-0.7	-4.3	-8.7
$\angle C_2C_3C_4O_5$(°)	0.0	-0.1	-26.0	53.9	0.0	0.1	0.0	-0.2	0.0	80.5	98.2	57.1
SOA (°)	364.4	371.4	358.6	353.0	357.3	357.4	358.1	355.5	355.4	371.3	361.3	344.4

Table S3: SA3(and SA2)-XMS(Im=0.3)-CASPT2(10,8)/cc-pVDZ (SSSR) energies (eV) and important geometrical parameters for critical points optimized at the aforementioned level for AcAc. Energies are reported relative to the ground state energy at the FC point (Staggered). MECIs are not confirmed to be minima on the intersection seam.

State/geom	FC (S)	S_1-min (E_d)	S_1-TS-Tw	T_1-min	asym-HTI-E_d	asym-HTI-S	asym-HTI-E_{up}	sym-HTI-E_d	sym-HTI-E_{up}	S_2/S_1-Tw	S_1/S_0-Tw	S_1/S_0-CI_{PyrC}
S_0	0.000	0.569	1.239	1.919	0.267	0.261	0.310	0.277	0.341	2.812	3.320	3.804
S_1	4.340	3.766	4.030	4.300	4.358	4.398	4.410	4.430	4.525	4.271	3.320	3.806
S_2	4.747	5.113	4.822	4.874	4.358	4.398	4.410	4.430	4.525	4.272	4.795	6.161
T_1	3.763	3.754	3.544	3.320	3.541	3.570	3.571	3.419	3.519	3.817	3.376	3.855
T_2	4.150	3.873	4.351	4.867	4.261	4.281	4.309	4.246	4.337	4.374	4.804	6.108
h_1 (Å)	1.011	0.973	0.990	0.980	1.066	1.059	1.046	1.201	1.195	0.971	0.972	1.002
h_2 (Å)	1.603	1.926	1.743	1.984	1.454	1.465	1.499	1.203	1.196	3.106	3.316	1.713
HT (Å)	-0.591	-0.953	-0.753	-1.004	-0.388	-0.406	-0.453	-0.002	0.000	-2.134	-2.344	-0.711
O_1-C_2 (Å)	1.255	1.352	1.294	1.244	1.309	1.312	1.311	1.336	1.343	1.308	1.236	1.250
C_4-O_5 (Å)	1.331	1.369	1.354	1.373	1.328	1.330	1.333	1.336	1.343	1.383	1.384	1.357
C_2-C_3 (Å)	1.456	1.405	1.422	1.458	1.450	1.452	1.446	1.440	1.433	1.376	1.468	1.469
C_3-C_4 (Å)	1.382	1.399	1.456	1.499	1.474	1.466	1.472	1.440	1.433	1.469	1.473	1.526
O_1-O_5 (Å)	2.542	2.745	2.578	2.805	2.477	2.480	2.497	2.370	2.355	3.490	3.617	2.625
$PyrC_3$ (°)	0.0	0.3	-7.7	8.2	0.0	-0.1	0.0	0.0	0.0	2.1	0.7	45.8
$\angle C_2C_3C_4$(°)	119.7	126.2	120.9	119.9	120.8	121.0	121.2	119.3	119.3	125.0	121.9	116.7
$\angle O_1C_2C_3C_4$(°)	0.0	1.7	3.5	-9.1	0.0	0.3	0.1	0.1	0.0	-0.1	-6.1	-11.2
$\angle C_2C_3C_4O_5$(°)	0.0	1.3	-25.3	54.2	0.0	0.1	0.0	0.0	0.0	78.6	99.0	55.6
SOA (°)	363.9	370.2	359.1	352.8	357.1	357.4	358.1	355.0	355.0	369.9	360.6	342.5

Table S4: SA3(and SA2)-XMS(Re=0.3)-CASPT2(14,12)/cc-pVDZ (MSMR) energies (eV) and important geometrical parameters for critical points optimized at the aforementioned level for MA. Energies are reported relative to the ground-state energy at the FC point. MECIs are not confirmed to be minima on the intersection seam.

State/geom	FC	S_1-min	S_1-TS-Tw	T_1-min	asym-HTI	sym-HTI	S_2/S_1-Tw	S_1/S_0-Tw	S_1/S_0-CI_{PyrC}
S_0	0.000	0.459	1.600	0.484	0.322	0.335	3.023	3.169	3.558
S_1	3.983	3.518	3.895	3.892	4.253	4.268	4.046	3.169	3.568
S_2	4.717	5.131	4.817	4.595	4.253	4.268	4.047	4.392	5.513
T_1	3.499	3.408	3.419	3.185	3.354	3.377	3.599	3.277	3.664
T_2	3.777	3.460	3.419	3.791	4.095	4.074	4.137	4.478	5.571
h_1 (Å)	1.002	0.968	1.004	0.993	1.126	1.216	0.971	0.972	1.001
h_2 (Å)	1.668	2.132	1.691	1.760	1.353	1.216	3.926	3.427	1.948
HT (Å)	-0.666	-1.164	-0.687	-0.768	-0.228	0.000	-2.955	-2.455	-0.947
O_1-C_2 (Å)	1.246	1.322	1.295	1.281	1.314	1.326	1.305	1.238	1.249
C_4-O_5 (Å)	1.327	1.360	1.343	1.351	1.321	1.326	1.377	1.371	1.341
C_2-C_3 (Å)	1.452	1.417	1.416	1.413	1.446	1.441	1.377	1.452	1.455
C_3-C_4 (Å)	1.378	1.411	1.465	1.479	1.459	1.441	1.463	1.465	1.489
O_1-O_5 (Å)	2.585	2.901	2.560	2.668	2.444	2.397	4.032	3.627	2.794
$PyrC_3$ (°)	0.0	0.2	-10.1	-1.7	0.0	0.0	1.6	-0.4	40.0
$\angle C_2C_3C_4$(°)	118.8	127.1	118.1	123.4	118.9	118.1	125.9	121.9	117.6
$\angle O_1C_2C_3C_4$(°)	0.0	1.3	4.4	-1.3	0.0	0.0	-0.4	3.0	-9.9
$\angle C_2C_3C_4O_5$(°)	0.0	2.7	-31.2	12.6	0.0	0.0	-110.6	-94.3	60.5
SOA (°)	366.6	375.7	357.6	364.2	357.5	356.8	377.2	367.0	349.6

Table S5: SA3(and SA2)-XMS(Im=0.3)-CASPT2(10,8)/cc-pVDZ (SSSR) energies (eV) and important geometrical parameters for critical points optimized at the aforementioned level for MA. Energies are reported relative to the ground state energy at the FC point. MECIs are not confirmed to be minima on the intersection seam.

State/geom	FC	S_1-min	S_1-TS-Tw	T_1-min	asym-HTI	sym-HTI	S_2/S_1-Tw	S_1/S_0-Tw	S_1/S_0-CI_{PyrC}
S_0	0.000	0.496	1.495	1.302	0.296	0.295	3.038	3.206	3.576
S_1	4.069	3.621	3.955	4.325	4.325	4.361	4.063	3.206	3.584
S_2	4.710	5.114	4.870	4.570	4.325	4.361	4.063	4.530	5.645
T_1	3.561	3.551	3.437	3.225	3.387	3.260	3.685	3.274	3.670
T_2	3.855	3.630	4.346	4.327	4.156	4.121	4.230	4.528	5.616
h_1 (Å)	1.007	0.972	0.994	0.983	1.100	1.212	0.971	0.972	0.992
h_2 (Å)	1.645	2.004	1.741	1.905	1.396	1.213	3.927	3.428	1.890
HT (Å)	-0.638	-1.031	-0.747	-0.922	-0.296	-0.001	-2.956	-2.456	-0.898
O_1-C_2 (Å)	1.249	1.345	1.287	1.249	1.307	1.326	1.304	1.234	1.244
C_4-O_5 (Å)	1.326	1.361	1.344	1.359	1.319	1.326	1.375	1.374	1.358
C_2-C_3 (Å)	1.451	1.404	1.418	1.437	1.451	1.442	1.373	1.459	1.464
C_3-C_4 (Å)	1.377	1.396	1.460	1.498	1.467	1.442	1.464	1.468	1.476
O_1-O_5 (Å)	2.569	2.811	2.574	2.757	2.458	2.390	4.041	3.664	2.754
$PyrC_3$ (°)	0.0	-0.1	-9.9	3.5	0.0	0.0	1.0	-0.8	43.3
$\angle C_2C_3C_4$(°)	118.5	125.6	118.5	120.3	119.1	118.1	125.8	122.3	115.9
$\angle O_1C_2C_3C_4$(°)	0.0	-0.2	4.1	-6.6	0.0	0.0	-0.6	2.7	-9.6
$\angle C_2C_3C_4O_5$(°)	0.0	-0.2	-29.6	43.1	0.0	0.0	-111.8	-96.1	60.5
SOA (°)	366.1	373.6	358.7	357.8	357.5	356.3	377.0	366.6	348.0

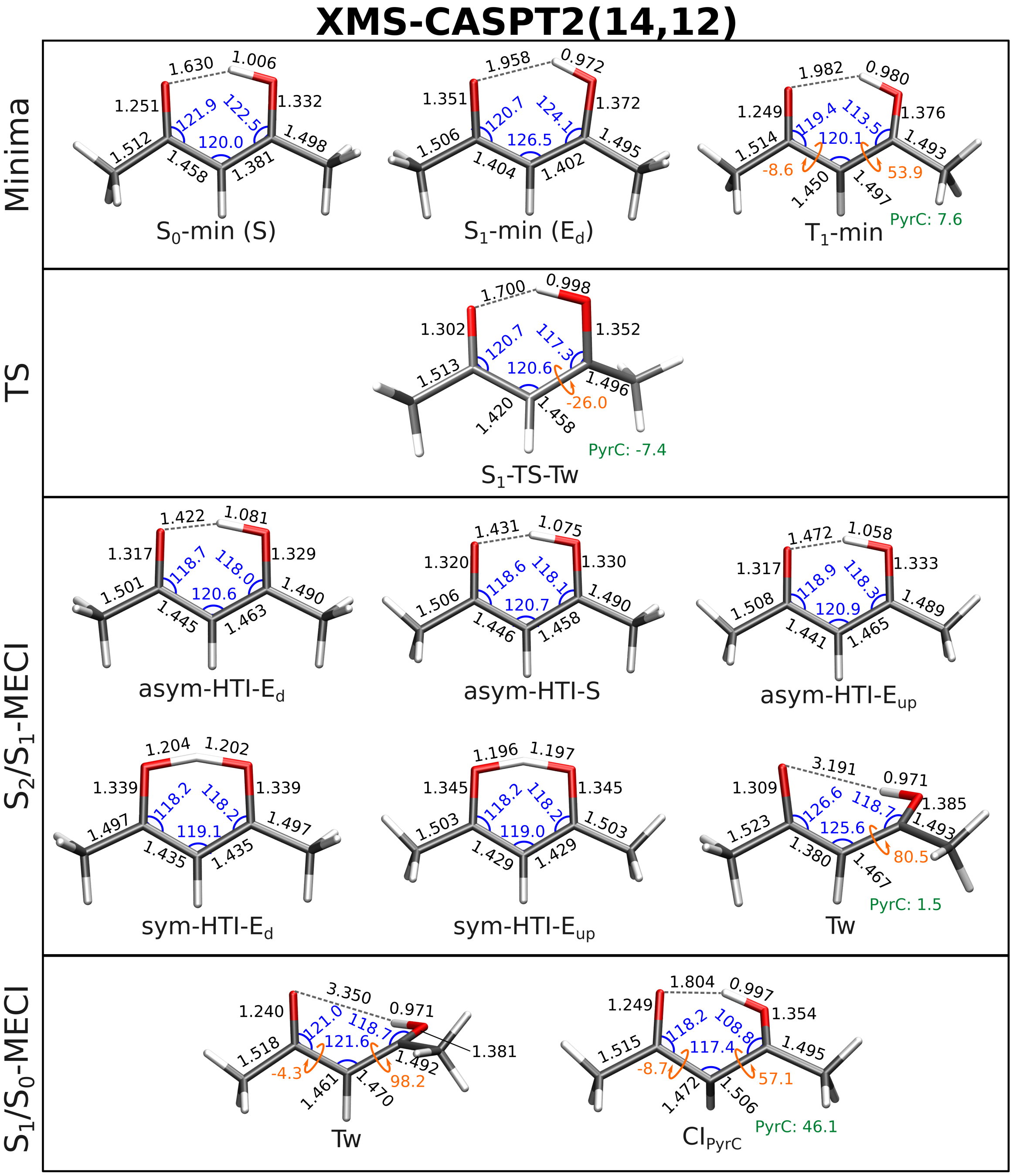

Figure S5: Critical point geometries for AcAc computed at the SA3/2-XMS(Re=0.3)-CASPT2(14,12)/cc-pVDZ (MSMR) level. S_1/S_0-CI_{PyrC} was obtained by extracting a representative hopping geometry from the dynamics and subsequently relaxing it to reach the nearby intersection seam. Hence, this point does not represent a MECI but rather a higher-energy point on the seam. Bond lengths in Å and angles in degrees. Dihedral angles <1° are not shown.

S3 Active-space stability and trajectory monitoring

Trajectory quality was assessed by monitoring energy conservation and population stability throughout the dynamics. Trajectory acceptance criteria were based on SHARC[14] defaults but with modestly relaxed thresholds (Table S6) because internally contracted XMS-CASPT2 (as implemented in BAGEL) is prone to larger energy fluctuations than uncontracted variants.[15,16] We verified on several trajectories that these adjusted thresholds did not cause spurious behaviors. With these criteria, 200 out of 256 trajectories for MA and 203 out of 265 trajectories for AcAc completed successfully.

Table S6: Trajectory monitoring criteria and associated thresholds used in the FSSH simulations.

Criterion	SHARC default	Threshold used
Max permissible drift in E_{tot} (eV)	0.2	0.5
Max permissible ΔE_{tot} (eV) between successive time steps	0.1	0.5
Max permissible active state ΔE_{pot} (eV) between successive time steps	0.7	0.7
Max permissible ΔE_{kin} (eV) between successive time steps	0.7	0.7
Max drift in total population	10^{-7}	10^{-7}
Max change in active state energy difference (eV) during a surface hop	1.0	1.0

We analyzed the failed trajectories to assess whether their exclusion might bias the population dynamics and geometric distributions. The majority (43/56 in MA, 58/62 in AcAc) of trajectory failures occurred after transfer to S_1, see Figure S6. As a result, the ground-state repopulation including all trajectories is ∼5% lower than when considering only successful ones (Figure S7). The geometric parameters of failed trajectories (shown at the failure time) fall within the distributions of the successful ones (Figure S9,Figure S10). However, a recurring feature among the failed trajectories was that after transfer to S_1, the enolic H-atom moved out of the molecular plane as the chelate ring expanded. This motion causes an artificial destabilization of the S_2 state at the SA3-XMS(Im=0.3)-CASPT2(10,8)/cc-pVDZ (SSSR) level, leading to energy jumps while leaving the character of S_1 (and its nuclear

gradient) largely unaffected.

Figure S8 illustrates this behavior for AcAc along a representative trajectory that failed. Immediately following population transfer to S_1 where the system is still near the the HTI seam (around 33-43 fs), the S_2 and S_1 have mixed $n\pi^*/\pi\pi^*$ character. As the ring expands, the S_1 and S_2 energies remain smooth and acquire $n\pi^*$ and $\pi\pi^*$ character, respectively. This is captured by XMS(Re=0.3)-CASPT2(14,12)/cc-pVDZ (MSMR) level, irrespective of whether state-averaging is performed over three, four or five singlet states (Figure S8a-c). In contrast, with the smaller active space, SA3 and SA4 lead to a significant destabilization of S_2 as the enolic H-atom moves out of plane because the state acquires double-excitation character. SA5 restores smoothness and correct electronic character of the S_2 state in this region of configuration even with the smaller active space. However, SA5 underestimates the S_2/S_1-gap at the FC point by about ~0.2 eV relative to our XMS-CASPT2(14,12) reference. Given the focus on the earliest S_2/S_1 dynamics, and the S_2 destabilization occurs only after the system has already transferred to S_1, we chose SA3-XMS(Im=0.3)-CASPT2(10,8)/cc-pVDZ (SSSR) for the decoherence-corrected FSSH simulations.

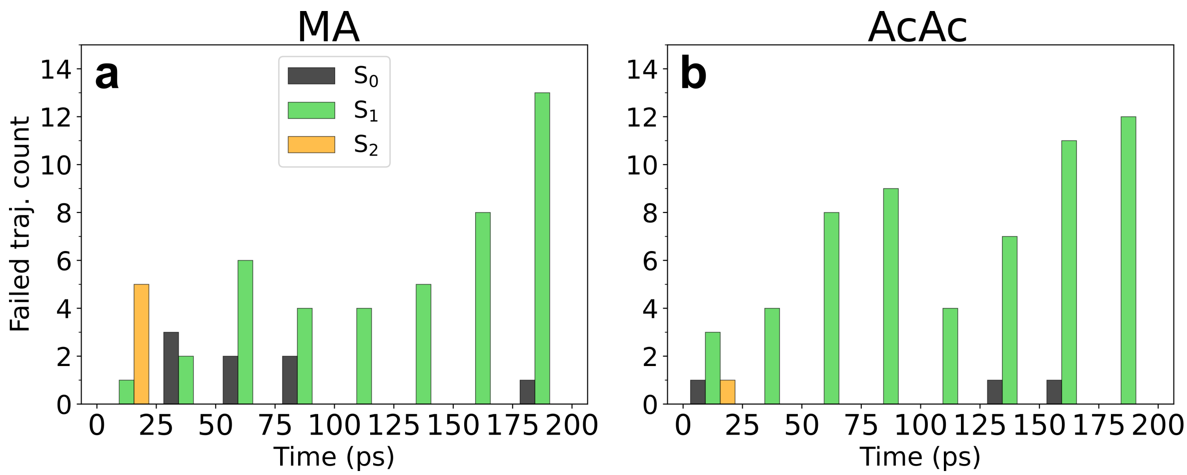

Figure S6: Failed trajectory counts per electronic state and over time (in 25-fs bins) for (a) MA and (b) AcAc.

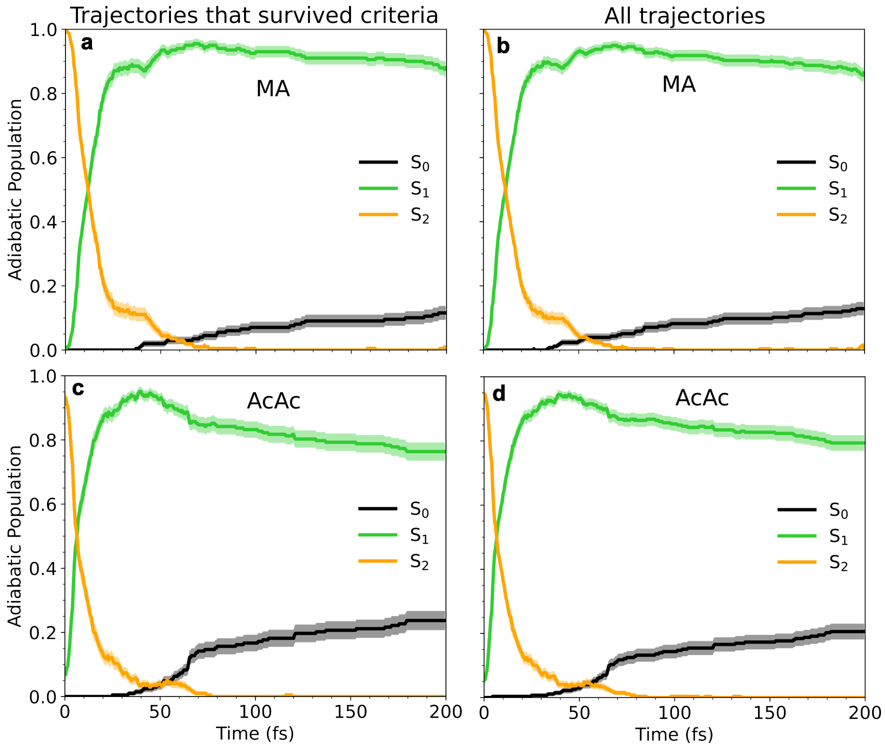

Figure S7: Comparison of adiabatic population dynamics for trajectories that survived up to 200 fs according to the above-mentioned criteria (a) for MA and (c) for AcAc) and all trajectories (including discarded trajectories)((b) for MA and (d) for AcAc). Shaded regions represent one bootstrap standard deviation obtained from 2000 samples. For AcAc, the majority (58/62) failed on S_1, which means that the S_0 population is slightly overestimated when excluding the failed trajectories.

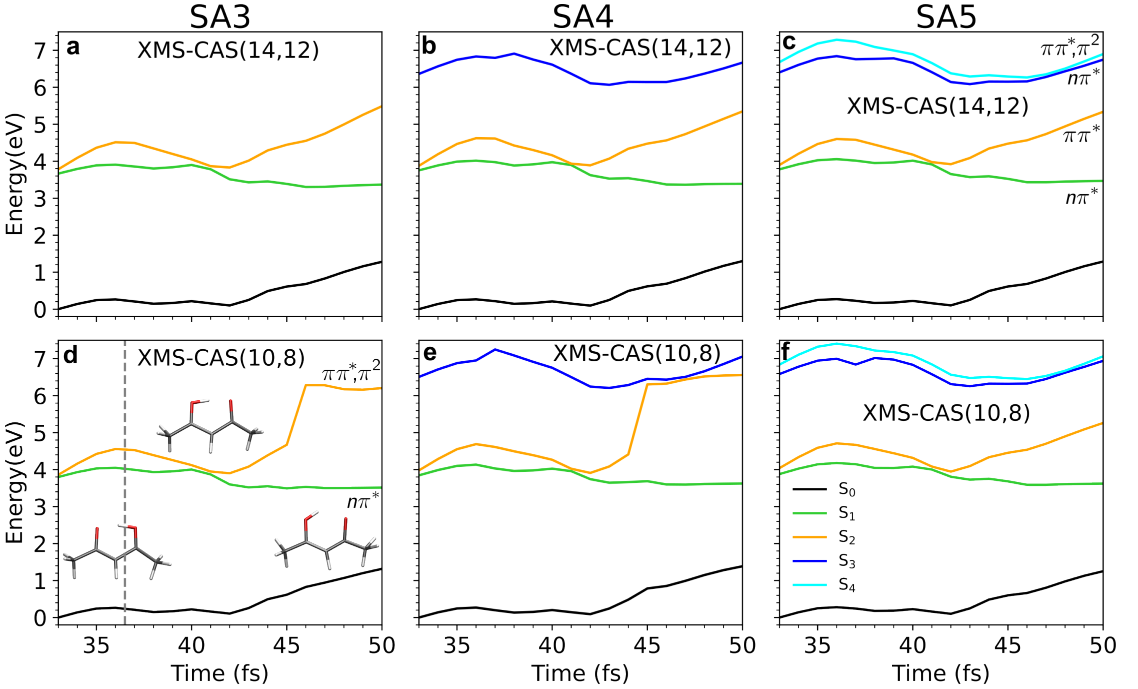

Figure S8: Comparison of excited states at the XMS(Re=0.3)-CASPT2(14,12)/cc-pVDZ (MSMR) and XMS(Im=0.3)-CASPT2(10,8)/cc-pVDZ (SSSR) levels, state-averaging over three, four and five states, respectively, between 32.5 fs to 50 fs of a representative failed trajectory. This trajectory was originally propagated at the SA3-XMS(Im=0.3)-CASPT2(10,8)/cc-pVDZ (SSSR) level. After hopping to S_1 surface at 32.5 fs (remains on S_1 surface from 33 fs (HT= −0.320 Å) and onwards), the trajectory traverses symmetric HTI regions (HT= −0.017 Å at 36.5 fs, shown by a dashed gray line in (d), after which the H is transferred to the other the O-atom (HT=0.369 Å at 40 fs) on S_1 state, and thereafter, the H-chelate ring starts to expand with the O-H group going in-and-out of the plane (HT=1.032 Å at 50 fs). Energies are reported relative to the S_0 energy at 33 fs, at the respective level of theory.

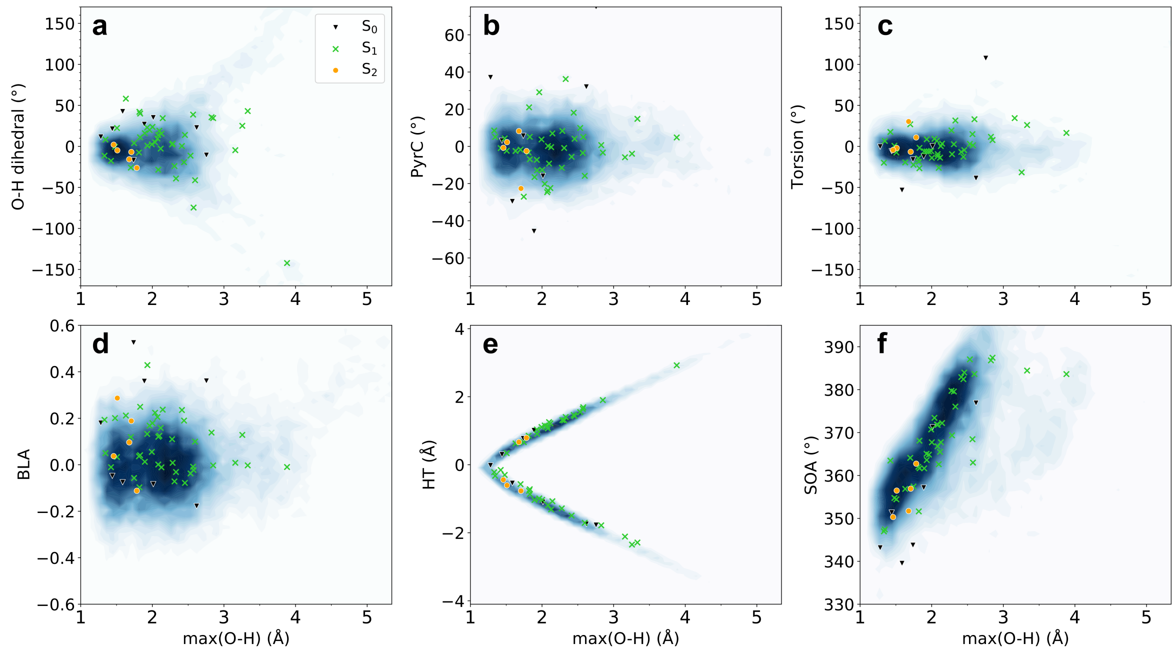

Figure S9: Geometric parameters of failed trajectories (S_0: black triangles, S_1: green crosses, S_2: orange circles) for MA at the time of failure compared to the corresponding distribution for the completed trajectories (blue filled contour). While we did observe a tendency on S_1 related to out-of-plane motion of the enolic H-atom, we did not find a distinct behavior that would suggest a biasing of the geometric distributions.

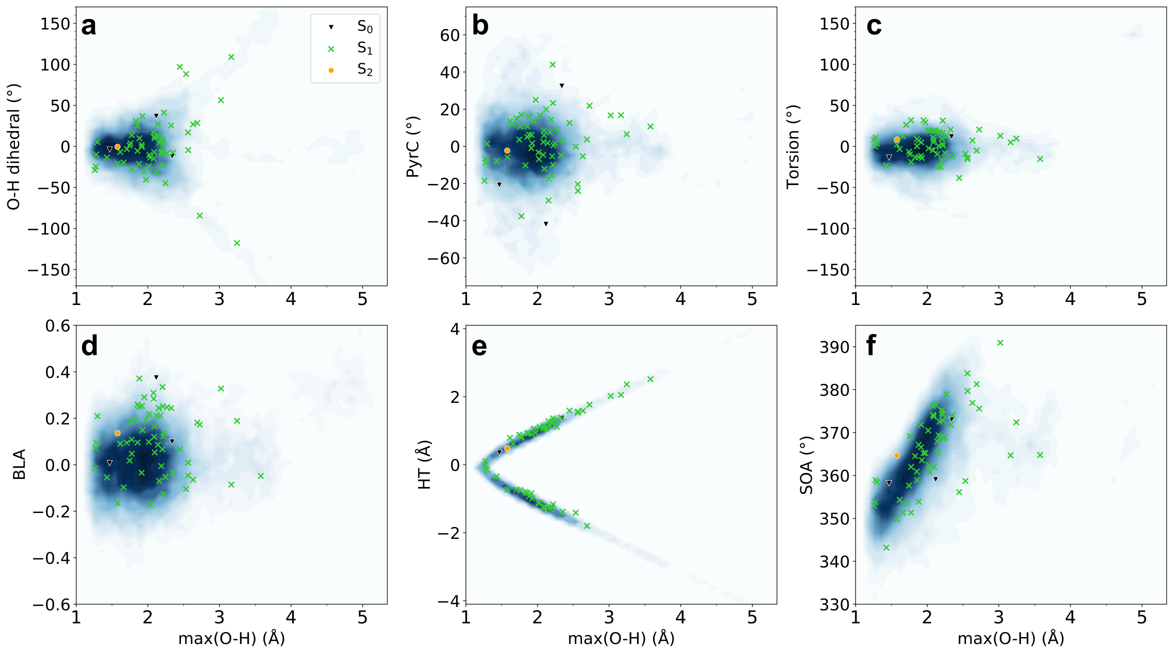

Figure S10: Geometric parameters of failed trajectories (S_0: black triangles, S_1: green crosses, S_2: orange circles) for AcAc at the time of failure compared to the corresponding distribution for the completed trajectories (blue filled contour). While we did observe a tendency on S_1 related to out-of-plane motion of the enolic H-atom, we did not find a distinct behavior that would suggest a biasing of the geometric distributions.

S4 Initial conditions for excited-state dynamics

In this section, we discuss the challenges related to generating initial conditions (ICs) for initiating trajectory-based nonadiabatic dynamics simulations of photoexcited AcAc and the approach taken in this work.

All ICs were generated using density-functional theory at the B3LYP(D3-BJ)/6-31G(d,p) level. While using the same electronic-structure method for IC sampling and nonadiabatic dynamics is often desirable, the present hybrid approach was chosen as an efficient and reasonable description of the ground-state vibrational distributions, which enabled the generation of a large ensemble of uncorrelated ICs from QT-AIMD. This choice was supported by the reasonable agreement between key structural parameters at the Franck–Condon geometry obtained with B3LYP(D3-BJ) and XMS-CASPT2(10,8), namely a BLA of -0.135 Å vs. -0.150 Å and a SOA coordinate of 363.5° vs. 363.9°, respectively, as well as an average deviation of -5.9 cm^{-1} in vibrational frequencies.

S4.1 Sampling approaches

Traditionally, IC sampling for trajectory-based excited-state dynamics is performed using one of two approaches:[17,18] (i) sampling from an approximate Wigner quasi-probability distribution[19] of the initial vibrational state; and (ii) classical Boltzmann sampling based on electronic ground-state *ab-initio* molecular dynamics (AIMD). The Wigner distribution naturally builds in nuclear quantum effects, but its practical use for molecules suffers from the harmonic approximation. In particular, linearized normal modes poorly represent low-frequency (floppy) modes that can cause exaggerated bond distances.[20,21] On the other hand, AIMD builds in anharmonic effects and has been used for larger, more flexible systems, including condensed phases, but at the expense of neglecting nuclear quantum effects.

Quantum thermostat *ab initio* molecular dynamics (QT-AIMD) was recently proposed as an alternative approach to IC sampling, aimed to alleviate these limitations.[21] Unlike

Boltzmann thermalization based on equipartitioning, QT thermalizes the normal modes of a molecule at their individual frequency-dependent temperatures using the generalized Langevin equation (GLE) thermostat.[22,23] In this way, QT produces the correct phase-space distribution in the limit of quantum harmonic oscillators. It was recently demonstrated that sampling ICs from a ground-state trajectory thermalized with QT-AIMD improves the description of flexible molecules with photoactive low-frequency normal modes, as reflected by more accurate photoabsorption cross-sections, quantum yields, and translational kinetic energy maps.[21]

S4.2 Challenges in IC-sampling of AcAc

The low-frequency methyl rotations in AcAc, particularly involving the keto-side methyl group, are poorly represented by harmonic modes, leading to artificially elongated C–H bonds. Such distorted geometries often cause convergence and energy conservation issues in CAS-based simulations due to the inadvertent inclusion of photochemically inactive C–H σ^* orbitals in the active space. In addition, the low methyl rotational barrier along with the shallow barrier to hydrogen transfer gives rise to multiple distinct stationary points on the ground-state potential energy surface.[24] These include both asymmetric and symmetric structures. We adopt the nomenclature introduced in Section S2. The stationary points are: asymmetric staggered minimum (S), asymmetric eclipsed-down (E_d), asymmetric eclipsed-up (E_{up}), asymmetric staggered opposite (S_s), symmetric eclipsed-down (E_{dHT}), symmetric eclipsed-up (E_{uHT}), symmetric staggered (E_{sHT}).

The nature of the ground-state vibrational wavefunction of AcAc remains debated.[25–29]Most electronic-structure studies identify the asymmetric C_s-structure (S) as the minimum when zero-point energy (ZPE) corrections are neglected.[30–35] Inclusion of ZPE favors the symmetric C_{2v}-structure (E_{dHT}).[35] Experimental results are mixed: microwave spectroscopy points to E_{dHT},[29] while electron diffraction has been interpreted as supporting either S[27,28] or E_{dHT}.[25,26] Recent simulations of the ground-state vibrational wavefunction with machine-

learned potentials have further highlighted this ambiguity. Approaches based on MP2 energies and gradients, employing permutationally invariant polynomials and Δ-machine learning with local CCSD(T) corrections, favor asymmetric eclipsed conformations.[24,36] By contrast, neural network PESs refined with thousands of local CCSD(T) energies via transfer-learning indicate a preference for the S structure.[37]

Because harmonic Wigner sampling struggles with both artificially long C–H bonds and the multiple low-barrier stationary points, it is not well-suited for AcAc. We therefore decided to employ QT-AIMD sampling. A detailed comparison between harmonic Wigner and QT-AIMD sampling for the two systems is provided in Section S4.4.

S4.3 Absorption spectrum

Figure S11 shows the simulated first absorption band for MA and AcAc, alongside the experimental spectrum of AcAc.[3] The simulated spectra were based on 1890 and 1784 geometries generated by QT-AIMD sampling. Oscillator strengths of the S_1 and S_2 states are shown as stick spectra. For S_2, the oscillator strength increases with excitation energy, reflecting reduced state-mixing as the S_2/S_1-energy gap widens. As shown in Figures S12 and S13, this gap variation correlates with distortions along the SOA, HT, and BLA coordinates, indicating that signatures of the HTI pathway are already imprinted in the IC-sampling in the FC region.

S4.4 QT-AIMD vs. harmonic Wigner sampling

To compare QT-AIMD and Wigner sampling, we generated 5000 ICs from a Wigner distribution at 298.15 K based on a vibrational analysis at the S_0-min geometry, obtained at the B3LYP(D3-BJ)/6-31G(d,p) level of theory.

We first examine the kinetic energy (KE) distributions (Figure S14). For QT-AIMD, we include all sampled geometries along the trajectory (all), the configurations used for absorption spectrum generation (abs), as well as the pump-pulse-selected configurations (pulse).

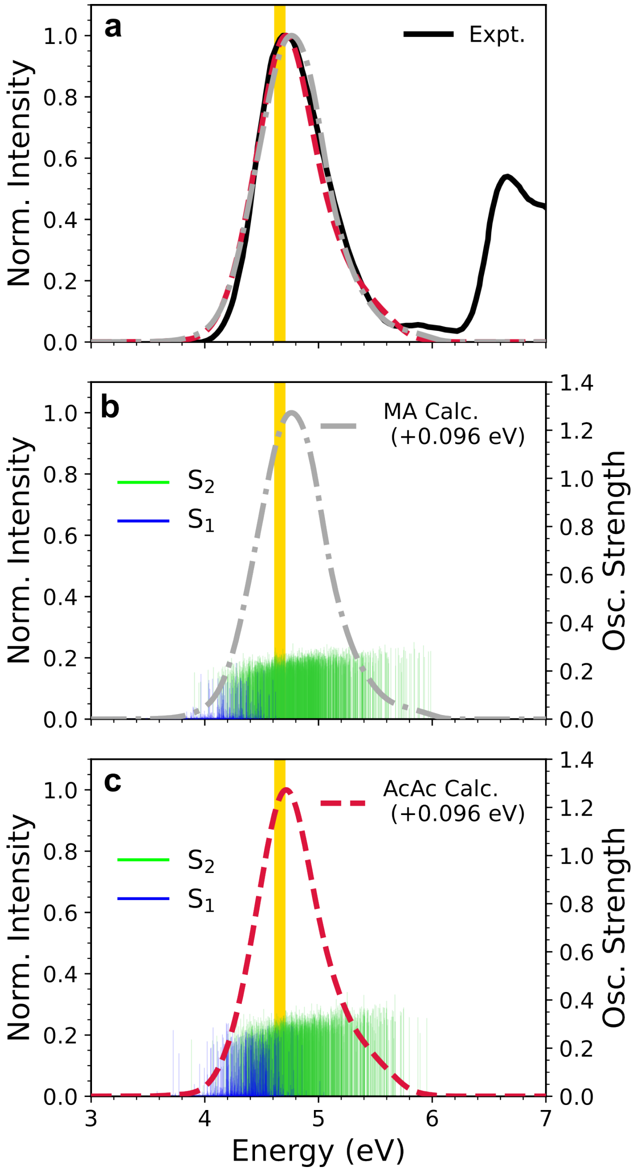

Figure S11: (a) Comparison of experimental and simulated electronic absorption spectra. The experimental spectrum of AcAc was digitized from Ref. 3, while the simulated spectra were obtained from QT-AIMD configurations (1890 geometries for MA and 1784 for AcAc) thermalized at 298.15 K at the B3LYP(D3-BJ)/6-31G(d,p) level. The yellow bar marks the pump window (4.661±0.05 eV) used to select initial conditions for excited-state dynamics. For AcAc (c), individual stick spectra were convolved with a Gaussian envelope (FWHM = 0.24 eV) and blue-shifted by +0.096 eV to align with experiment. For MA (b), where no experimental spectrum is available, the same convolution and shift were applied. Stick spectra include contributions from both S_2 and S_1, and for S_2 show a correlation between excitation energy and oscillator strength. The slightly larger oscillator strengths in AcAc relative to MA reflect a greater electronic delocalization due to the methyl groups.

For MA, QT-AIMD and Wigner sampling produce quite similar KE profiles, with maxima slightly below half the ZPE, consistent with the absence of low-frequency anharmonic modes. For AcAc, however, more pronounced differences emerge: Wigner-sampled ICs produce a KE distribution centered near half the ZPE, especially for geometries with average C–H distances <1.2 Å, whereas QT-AIMD shifts the maximum to lower KE due to anharmonic effects.

Geometric distributions further highlight these differences. Harmonic Wigner sampling generates displacements only around the minimum geometry, neglecting other low-energy regions of the PES. As pointed out above, this limitation is particularly pronounced in AcAc, where multiple stationary points are separated by shallow barriers. By contrast, QT-AIMD explores the ground-state landscape to a larger extent. In Figure S15, QT-AIMD (a-c) visits all stationary points with highest density near the E_d-conformer, consistent with previous predictions,[24,36] while Wigner sampling (d) remains restricted to the S-conformer. Similarly, Figure S16 shows O–H transfer events in QT-AIMD, absent in room-temperature Wigner sampling. Wigner ICs with average C–H <1.2 Å (e) again bias toward the S-conformer, while QT-AIMD recovers broader configurational diversity. Finally, Figure S17 shows that all QT-AIMD schemes, and restricted Wigner sampling, reproduce average C–H distances near 1.1 Å, but total Wigner sampling is skewed toward elongated bonds.

In summary, QT-AIMD provides a more realistic description of the flat ground-state PES of AcAc, capturing multiple stationary points, enabling O–H delocalization, and reproducing C-H bond lengths associated with low-frequency methyl torsions. Harmonic Wigner sampling, in contrast, remains overly localized around the minimum and fails to capture anharmonic effects.

S5 Additional analyses of the dynamics

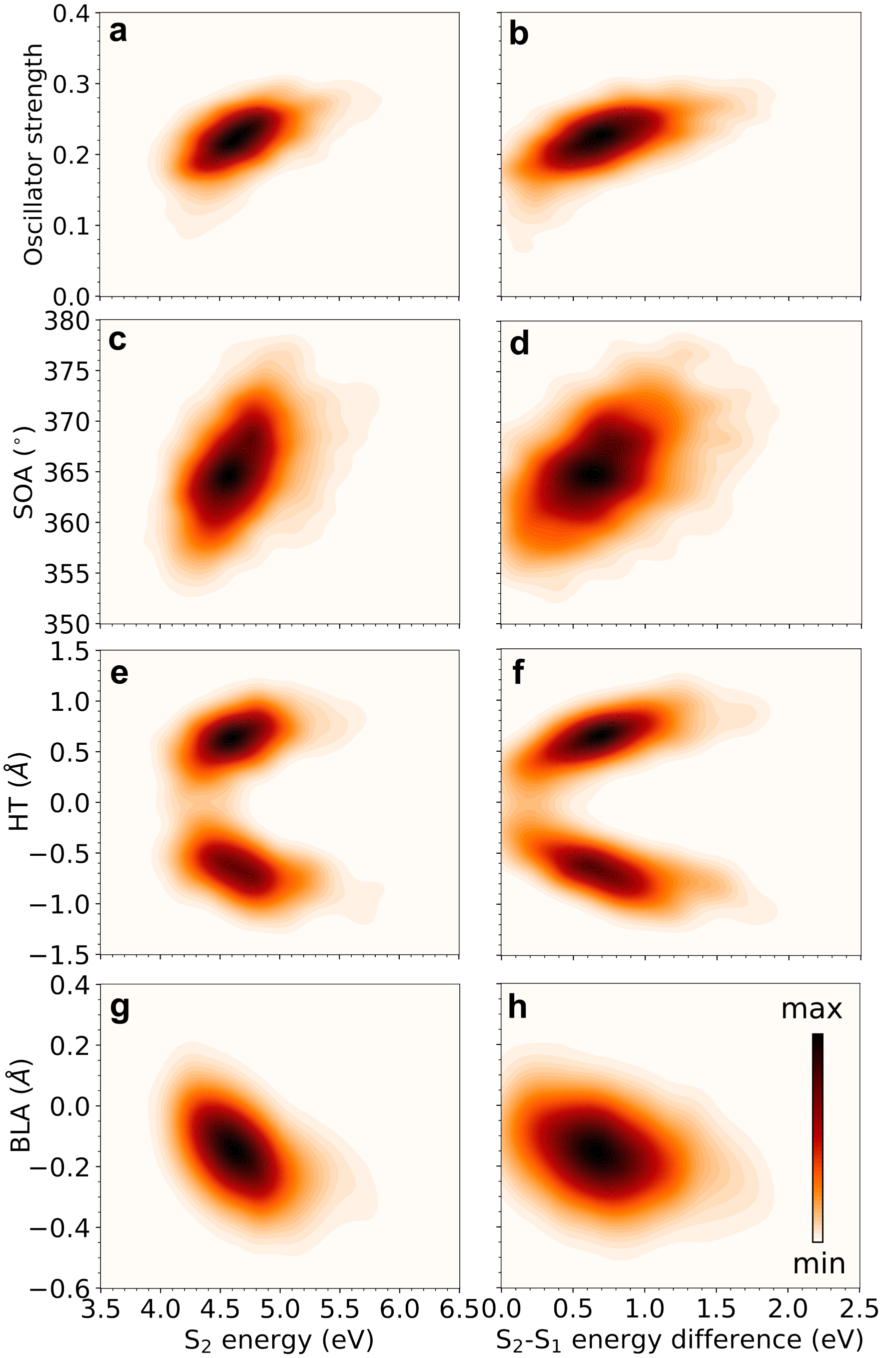

Figure S12: Correlation of oscillator strength and structural coordinates with the S_2 energy (a,c,e,g) and the S_2/S_1-energy gap (b,d,f,h) for MA. Shown are (a,b) S_2 oscillator strength, (c,d) SOA, (e,f) HT coordinate, and (g,h) BLA. Nonadiabatic dynamics and PES scans demonstrate that the S_2 and S_1 states approach each other along the HT coordinate, BLA, and contraction of the H-chelate ring; this figure illustrates that such trends are already encoded in the IC-sampling. The 2D-distributions were smoothed by convolution with a Gaussian kernel ($\sigma_x = 0.1$ eV; $\sigma_y = 0.01$, 1°, 0.1 Å, and 0.067 Å for a/b, c/d, e/f and g/h, respectively).

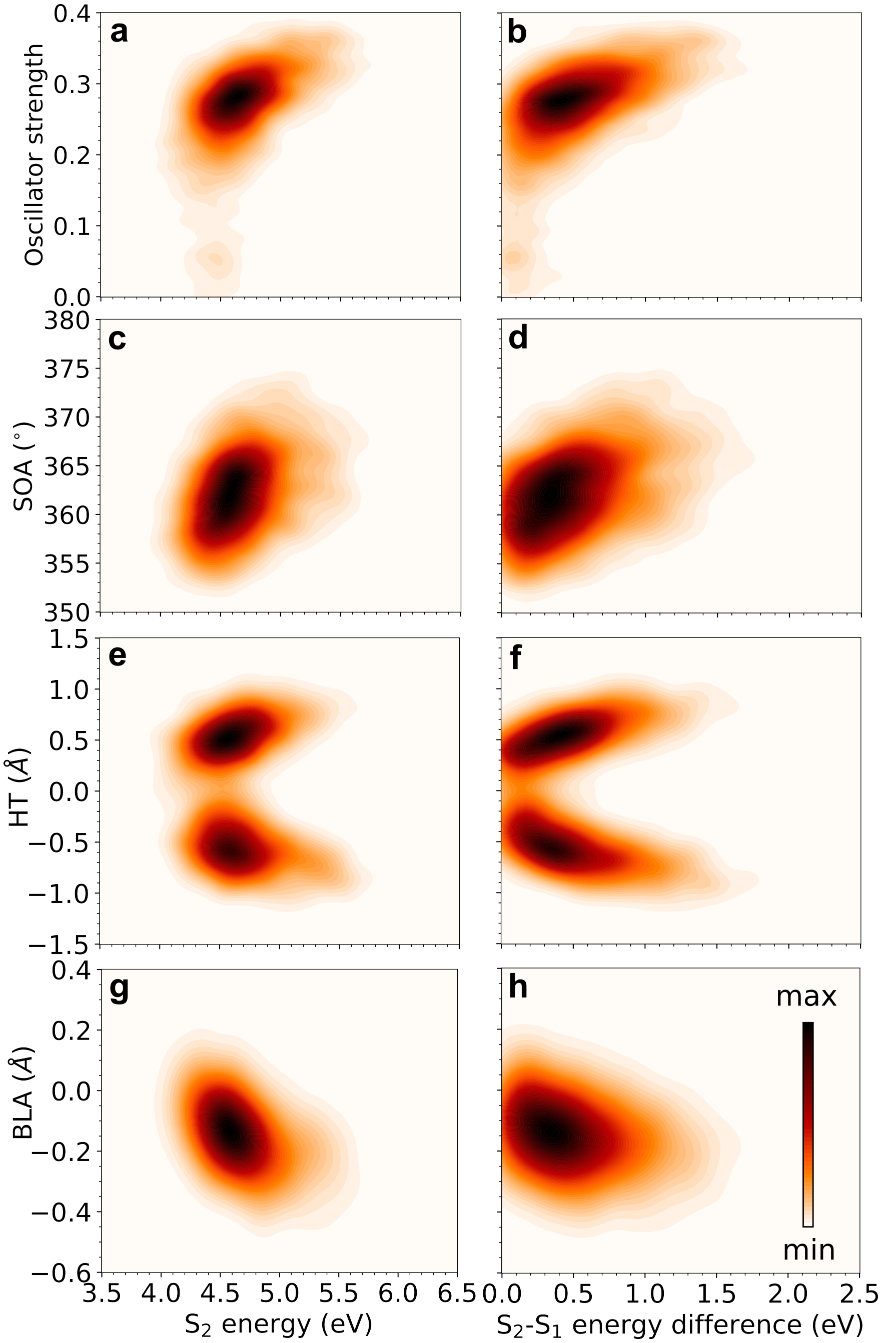

Figure S13: Correlation of oscillator strength and structural coordinates with the S_2 energy (a,c,e,g) and the S_2/S_1-energy gap (b,d,f,h) for AcAc. Shown are (a,b) S_2 oscillator strength, (c,d) SOA, (e,f) HT coordinate, and (g,h) BLA. Nonadiabatic dynamics and PES scans demonstrate that the S_2 and S_1 states approach each other along the HT coordinate, BLA, and contraction of the H-chelate ring; this figure illustrates that such trends are already encoded in the IC-sampling. The 2D-distributions were smoothed by convolution with a Gaussian kernel ($\sigma_x = 0.1$ eV; $\sigma_y = 0.01$, 1°, 0.1 Å, and 0.067 Å for a/b, c/d, e/f and g/h, respectively).

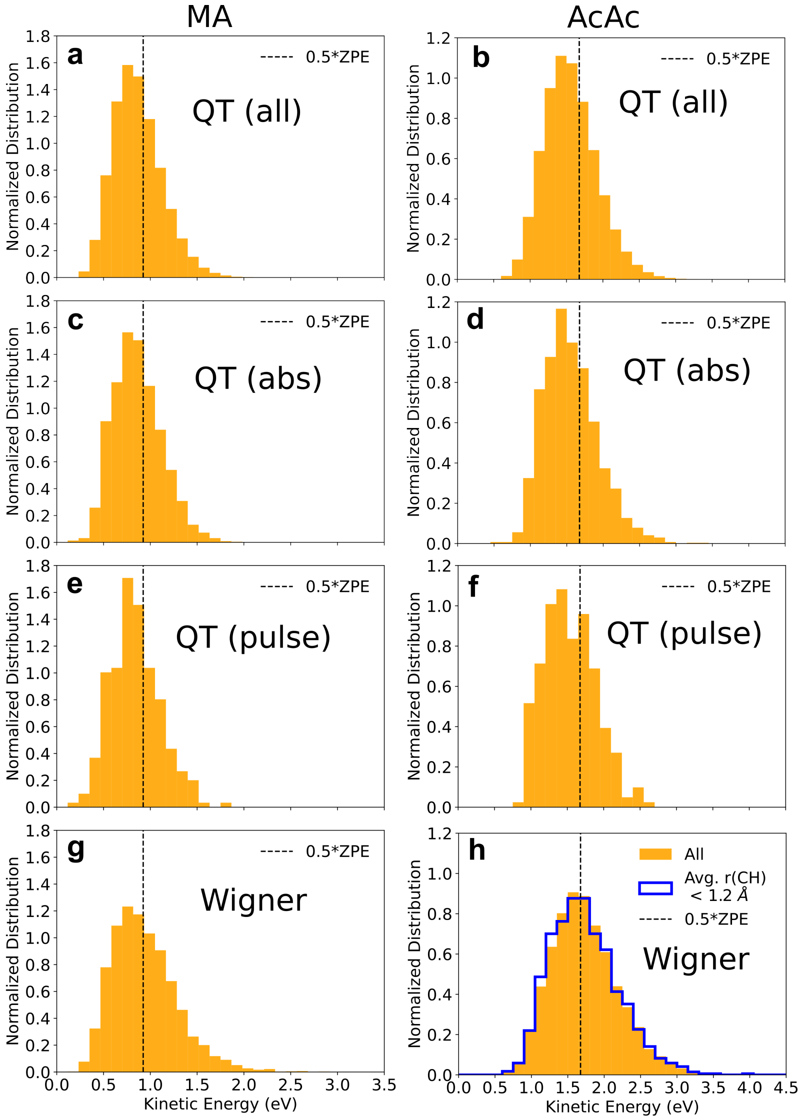

Figure S14: Kinetic energy (KE) distributions from (a-f) QT-AIMD sampling, and (g, h) harmonic Wigner sampling in MA and AcAc. For QT-AIMD sampling, (a,b) include all geometries from the thermalized trajectories, (c,d) the subsets of 1890 (MA) and 1784 (AcAc) ICs used for absorption spectrum generation (Gaussian broadening, FWHM = 0.24 eV), and (e,f) the 256 (MA) and 265 (AcAc) ICs selected within a 0.10 eV window around the pump photon energy (4.661 eV, 266 nm). In MA, both QT-AIMD and Wigner-sampled KE distributions peak below half of the ZPE. In AcAc, the Wigner distribution is centered near half of the ZPE, whereas QT sampling shifts the maximum to lower KE due to anharmonicity.

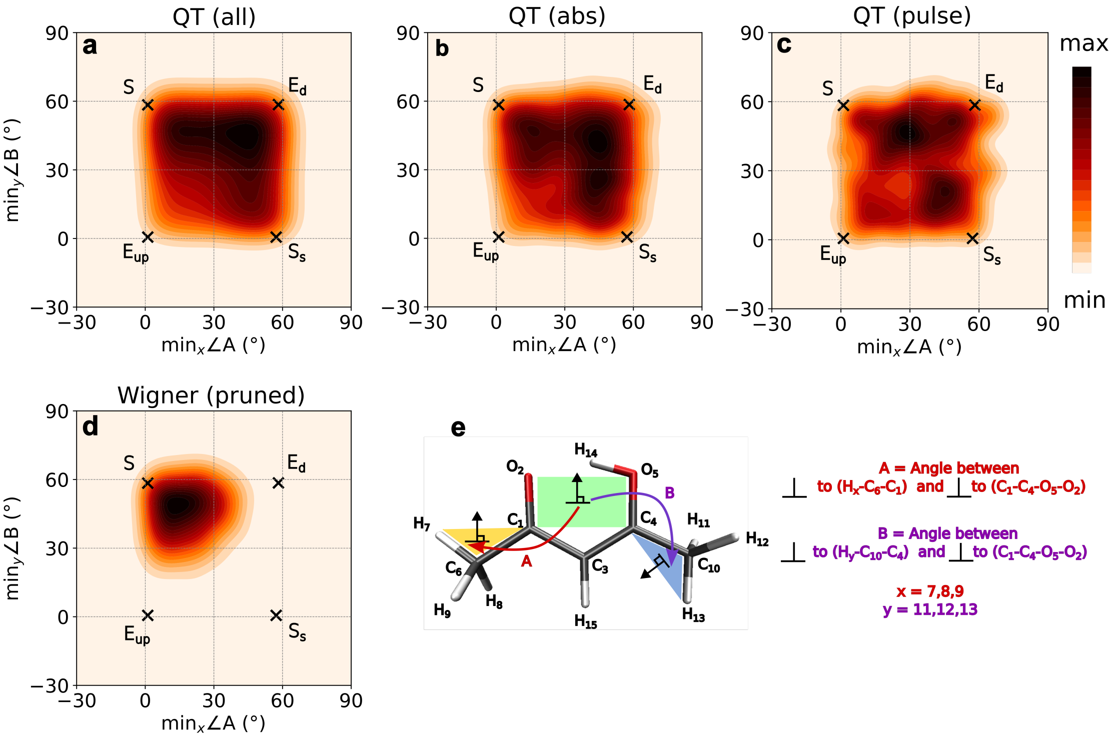

Figure S15: Comparison of QT-AIMD and harmonic Wigner sampling for AcAc at 298.15 K (B3LYP(D3-BJ)/6-31G(d,p)). QT-AIMD sampling: (a) all geometries from the thermalized trajectory, (b) 1784 ICs sampled every 710 fs from this trajectory for absorption spectrum generation; (c) 265 ICs selected within a 0.10 eV window around the pump photon energy, (d) ICs from a harmonic Wigner distribution (restricted to average C–H distance <1.2 Å) based on the S-conformer. (e) Definition of angles A and B. Staggered minimum (S), eclipsed-down (E_d), eclipsed-up (E_{up}), and staggered saddle point (S_s) are marked with black crosses at the following coordinates: (1.1°, 58.4°), (58.2°, 58.5°), (1.1°, 0.6°), and (57.2°, 0.6°), respectively. The remaining stationary points S_{sHT}, E_{dHT}, and E_{uHT} coincide approximately with S_s, E_d, and E_{up} and are therefore not shown explicitly. The 2D-distributions were convolved with a Gaussian kernel ($\sigma = 5°$ in both directions).

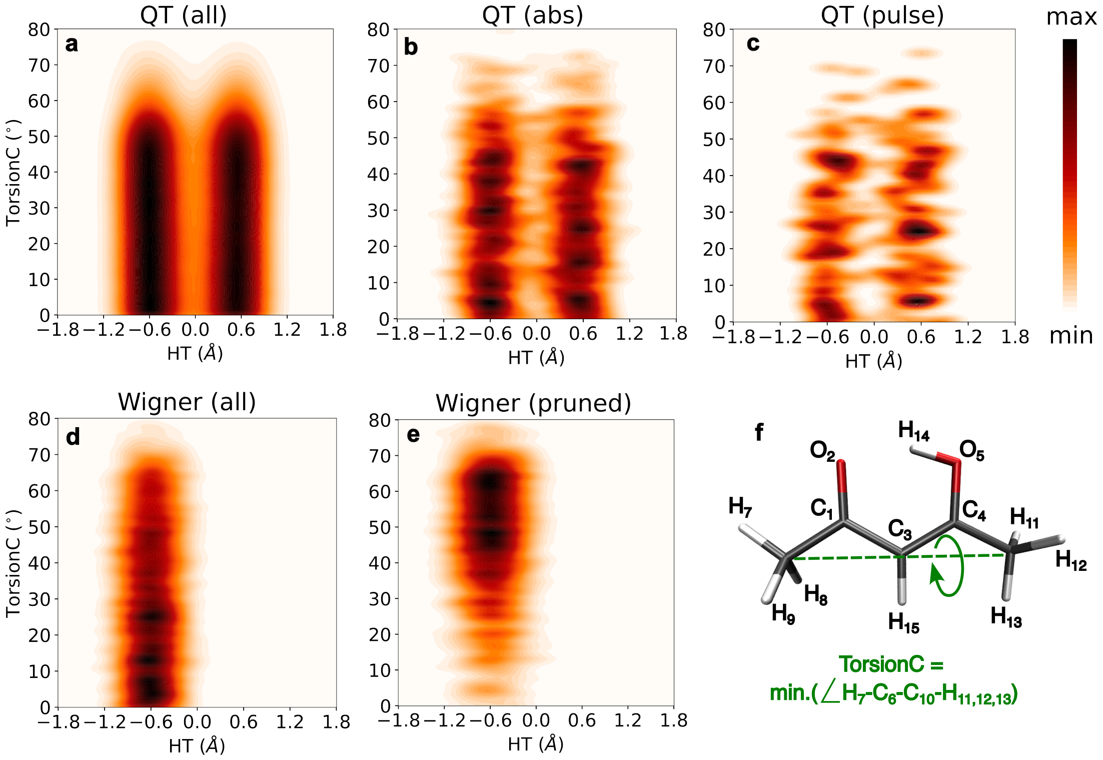

Figure S16: Distribution of the HT-coordinate with respect to TorsionC from QT-AIMD and harmonic Wigner sampling of AcAc at 298.15 K (B3LYP(D3-BJ)/6-31G(d,p)). QT-AIMD sampling: (a) all geometries from the thermalized trajectory, (b) 1784 ICs sampled every 710 fs for absorption spectrum generation, and (c) 265 ICs selected within a 0.10 eV window around the pump photon energy. Wigner sampling: (d) all 5000 initial conditions, and (e) subset with average C–H distance $<$1.2 Å. (f) Definition of TorsionC ($\sim$60° = staggered, $\sim$0° = eclipsed). QT-AIMD produces a bimodal distribution along the HT-coordinate, showing that H-transfer structures are accessible already in the ground state. In contrast, Wigner sampling yields a unimodal HT-distribution restricted to the staggered minimum; even when selecting ICs with short C–H distances (e), the distribution remains dominated by the S-conformer. The 2D-distributions were smoothed by Gaussian convolution ($\sigma_x = 0.15$ Å and $\sigma_y = 1°$).

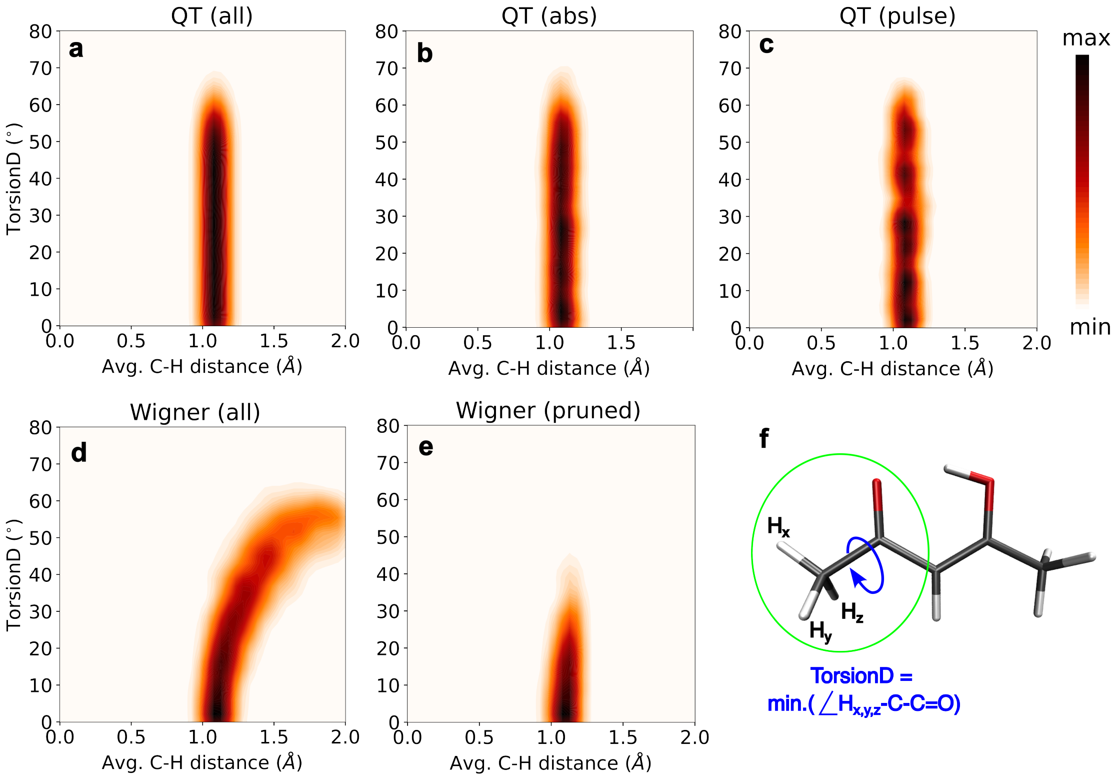

Figure S17: Distribution of TorsionD relative to the average methyl C–H distance for QT-AIMD and harmonic Wigner sampling of AcAc at 298.15 K (B3LYP(D3-BJ)/6-31G(d,p)). QT-AIMD sampling: (a) all geometries from the thermalized trajectory, (b) 1784 ICs sampled every 710 fs for absorption spectrum generation, and (c) 265 ICs selected within a 0.10 eV window around the pump photon energy. Wigner sampling: (d) all 5000 initial conditions, and (e) subset with average C–H distance <1.2 Å. The TorsionD coordinate is defined in (f). Wigner sampling shows large average C–H bond lengths due to the low-frequency methyl rotation ($\sim$48 cm^{-1}). Pruning the Wigner samples to only include geometries with average C–H distance <1.2 Å restricts sampling to be around the S-conformer. On the other hand, QT-AIMD allows for improved sampling of methyl rotations. The 2D-distributions were convolved with a Gaussian ($\sigma = 0.05$ Å along HT, and 2° along TorsionD).

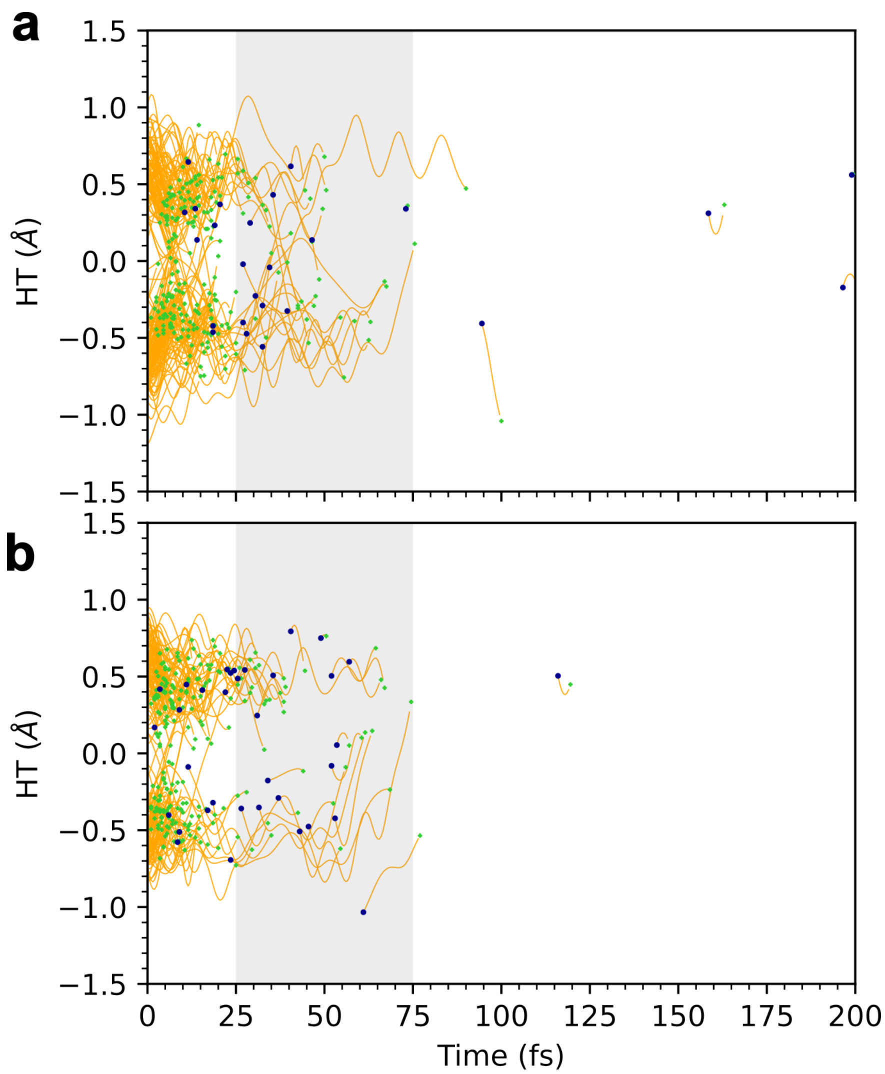

Figure S18: HT-coordinate of trajectories on the S_2 state for (a) MA and (b) AcAc. The gray shaded area between 25-75 fs shows that there is still a small fraction trajectories on the S_2 surface near the S_2/S_1-HTI region for both molecules. S_2/S_1-forward hops and -backward hops are represented by green and blue markers, respectively.

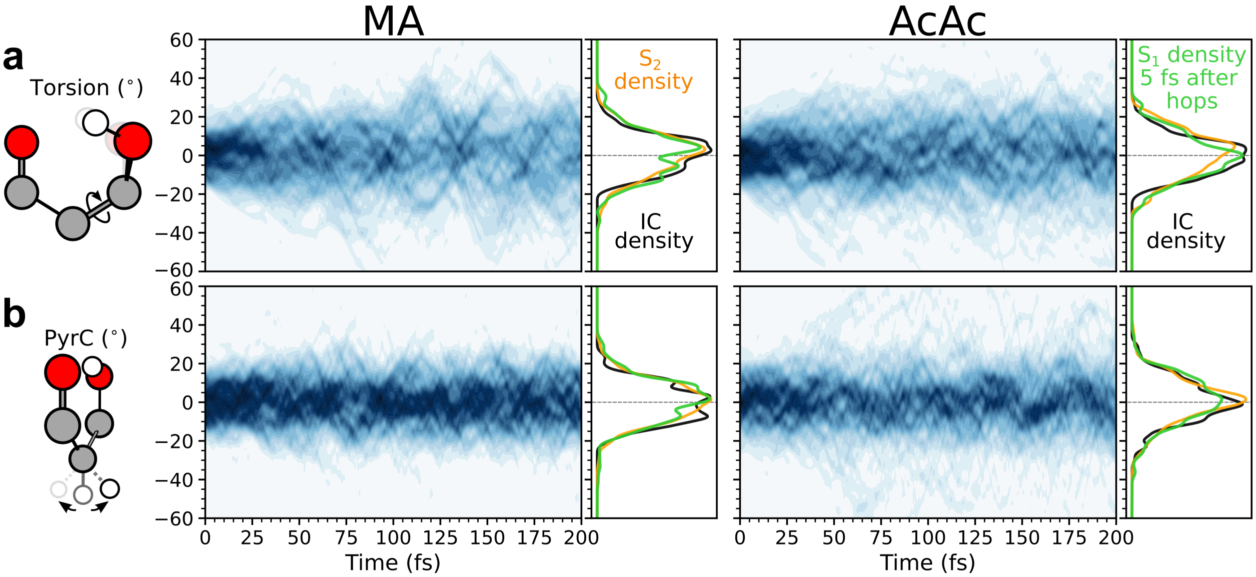

Figure S19: Time evolution of the nuclear density along the (a) Torsion and (b) PyrC coordinates within 200 fs after photoexcitation for (left column) MA and (right column) AcAc. The 1D-distributions on the right show how the nuclear distribution changes from the FC region and up to 5 fs after population transfer to S_1: black lines represent the IC distribution; orange lines the integrated S_2 density; and green lines the S_1 density integrated over the initial 5 fs after the S_2/S_1-hop. The reduced nuclear densities were generated by Gaussian convolution along the respective geometric dimension ($\sigma_y = 2°$).

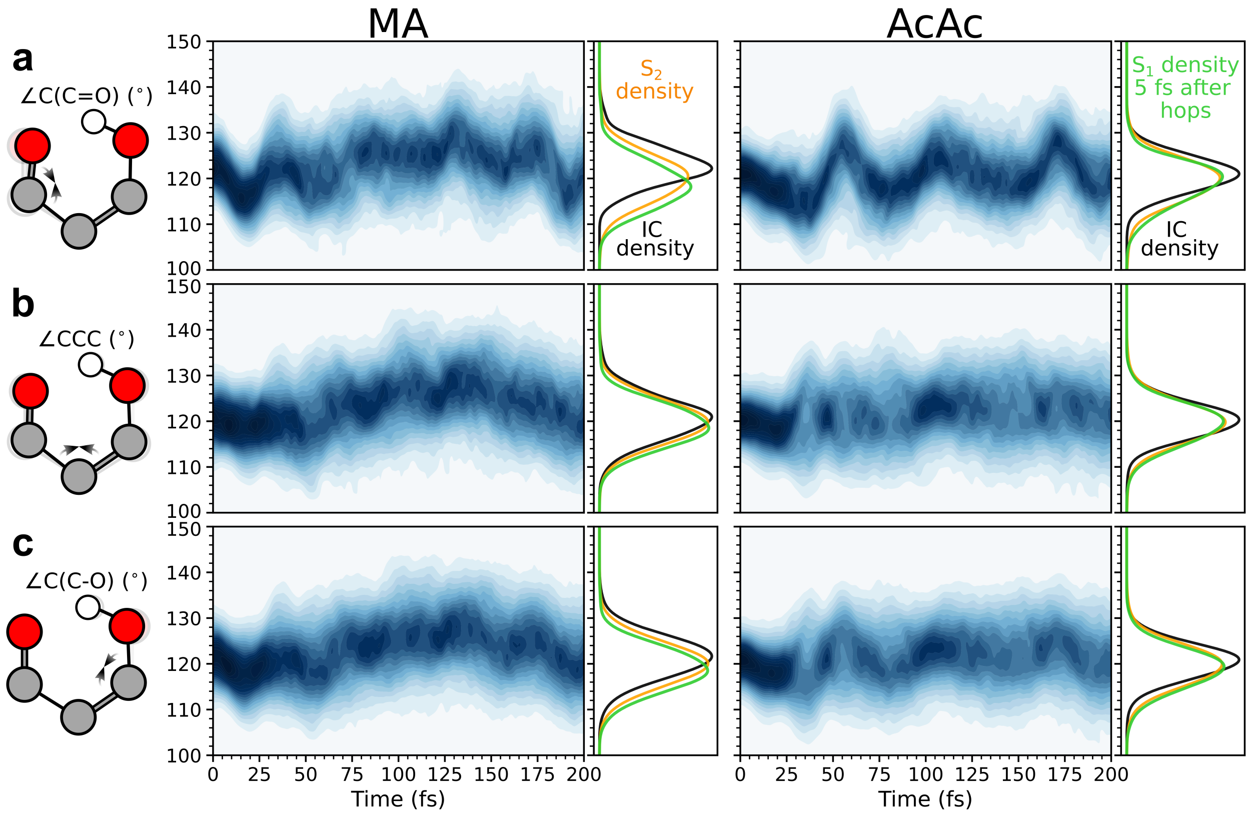

Figure S20: Time evolution of the nuclear density along the three angles composing the SOA coordinate (a) ∠C(C=O) (b) ∠CCC and (c) ∠C(C–O) coordinates within 200 fs after photoexcitation for (left column) MA and (right column) AcAc. The 1D-distributions on the right show how the nuclear distribution changes from the FC region and up to 5 fs after population transfer to S_1: black lines represents the IC distribution; orange lines the integrated S_2 density; and green lines the S_1 density integrated over the initial 5 fs after the S_2/S_1-surface hop. The reduced nuclear densities were generated by Gaussian convolution along the respective geometric dimension ($\sigma_y = 2°$).

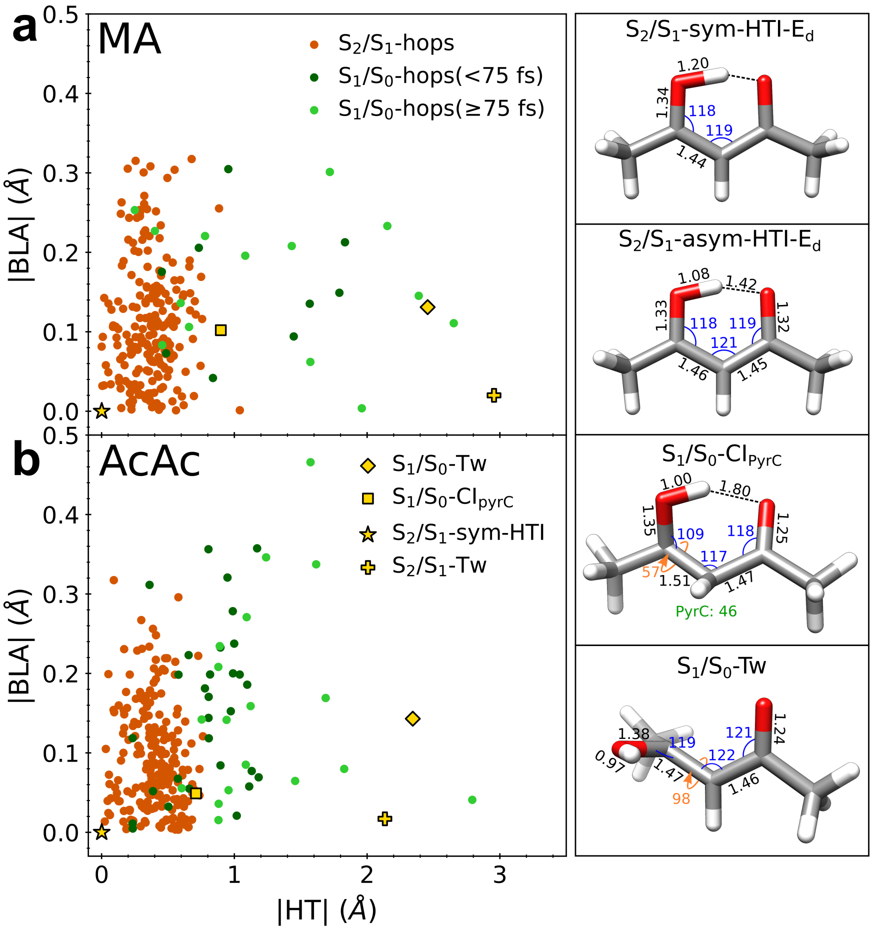

Figure S21: Differentiating the S_2/S_1-nonadiabatic transfer events (i.e., surface hops) from the early and late S_1/S_0-hopping events in the space spanned by PyrC and torsional modes for (a) MA and (b) AcAc. The S_2/S_1-hops are mediated by in-plane motion near the HTI region whereas the S_1/S_0-hops are mediated by out-of-plane motion. In AcAc, the early S_1/S_0-hops show a clear preference to be more centrally pyramidalized, with the later hops becoming increasingly twisted. Red, dark-green, and lime-green solid circles represent the S_2/S_1-hops, the early S_1/S_0-hops (<75 fs), and the late S_1/S_0-hops (≥75 fs), respectively. The golden markers represent the location of the MECIs. The structures for the dynamically important symmetric and asymmetric S_2/S_1-HTI-E_d, S_1/S_0-Tw and S_1/S_0-CI_{PyrC} intersection points for AcAc are shown on the right.

References

(1) Seliskar, C. J.; Hoffmann, R. E. $1^1B_1(n\pi^*) \leftarrow 1^1A_1$ transition of malondialdehyde. *Journal of the American Chemical Society* **1977**, *99*, 7072–7073, DOI: doi:10.1021/ja00463a056.

(2) Walzl, K. N.; Xavier, J., I. M.; Kuppermann, A. Electron-impact spectroscopy of various diketone compounds. *The Journal of Chemical Physics* **1987**, *86*, 6701–6706, DOI: doi:10.1063/1.452418.

(3) Nakanishi, H.; Morita, H.; Nagakura, S. Electronic Structures and Spectra of the Keto and Enol Forms of Acetylacetone. *Bulletin of the Chemical Society of Japan* **1977**, *50*, 2255–2261, DOI: doi:10.1246/bcsj.50.2255.

(4) Seliskar, C.; Hoffmann, R. The 3540-Å electronic transition of malonaldehyde, a tunneling hydrogen-bonded molecule. *Journal of Molecular Spectroscopy* **1981**, *88*, 30–40, DOI: doi:https://doi.org/10.1016/0022-2852(81)90351-9.

(5) Coe, J. D.; Martínez, T. J. Ab Initio Molecular Dynamics of Excited-State Intramolecular Proton Transfer around a Three-State Conical Intersection in Malonaldehyde. *The Journal of Physical Chemistry A* **2006**, *110*, 618–630, DOI: doi:10.1021/jp0535339, PMID: 16405334.

(6) Sobolewski, A. L.; Domcke, W. Photophysics of Malonaldehyde: An ab Initio Study. *The Journal of Physical Chemistry A* **1999**, *103*, 4494–4504, DOI: doi:10.1021/jp990030s.

(7) Sapunar, M.; Ayari, T.; Došlić, N. Comparative study of the photodynamics of malonaldehyde and acetylacetone. *Chemical Physics* **2018**, *515*, 622–627, DOI: doi:https://doi.org/10.1016/j.chemphys.2018.07.042, Ultrafast Photoinduced Processes in Polyatomic Molecules:Electronic Structure, Dynamics and Spectroscopy (Dedicated to Wolfgang Domcke on the occasion of his 70th birthday).

(8) Faber, R.; Kjønstad, E. F.; Koch, H.; Coriani, S. Spin adapted implementation of EOM-CCSD for triplet excited states: Probing intersystem crossings of acetylacetone at the carbon and oxygen K-edges. *J. Chem. Phys.* **2019**, *151*, 144107, DOI: doi:https://doi.org/10.1063/1.5112164.

(9) Tsuru, S.; Vidal, M. L.; Pápai, M.; Krylov, A. I.; Møller, K. B.; Coriani, S. An assessment of different electronic structure approaches for modeling time-resolved x-ray absorption spectroscopy. *Structural Dynamics* **2021**, *8*, 024101, DOI: doi:10.1063/4.0000070.

(10) Xie, B.; Cui, G.; Fang, W.-H. Multiple-State Nonadiabatic Dynamics Simulation of Photoisomerization of Acetylacetone with the Direct ab Initio QTMF Approach. *Journal of Chemical Theory and Computation* **2017**, *13*, 2717–2729, DOI: doi:10.1021/acs.jctc.7b00153, PMID: 28437107.

(11) Squibb, R. J.; Sapunar, M.; Ponzi, A.; Richter, R.; Kivimäki, A.; Plekan, O.; Finetti, P.; Sisourat, N.; Zhaunerchyk, V.; Marchenko, T.; Journel, L.; Guillemin, R.; Cucini, R.; Coreno, M.; Grazioli, C.; Di Fraia, M.; Callegari, C.; Prince, K. C.; Decleva, P.; Simon, M.; Eland, J. H. D.; Došlić, N.; Feifel, R.; Piancastelli, M. N. Acetylacetone photodynamics at a seeded free-electron laser. *Nature Communications* **2018**, *9*, 63, DOI: doi:10.1038/s41467-017-02478-0.

(12) List, N. H.; Dempwolff, A. L.; Dreuw, A.; Norman, P.; Martínez, T. J. Probing competing relaxation pathways in malonaldehyde with transient X-ray absorption spectroscopy. *Chem. Sci.* **2020**, *11*, 4180–4193, DOI: doi:10.1039/D0SC00840K.

(13) Severino, S.; Aleotti, F.; Mai, L.; Crego, A.; Medeghini, F.; Frassetto, F.; Poletto, L.; Lucchini, M.; Segatta, F.; Reduzzi, M.; Nisoli, M.; Nenov, A.; Borrego-Varillas, R. Mapping Excited-State Decay Mechanisms in Acetylacetone by Sub-20 fs Time-Resolved

Photoelectron Spectroscopy. *Journal of the American Chemical Society* **2025**, *147*, 30785–30793, DOI: doi:10.1021/jacs.5c06327, PMID: 40801839.

(14) Mai, S.; Marquetand, P.; González, L. Nonadiabatic dynamics: The SHARC approach. *WIREs Computational Molecular Science* **2018**, *8*, e1370, DOI: doi:https://doi.org/10.1002/wcms.1370.

(15) Pulay, P. A perspective on the CASPT2 method. *International Journal of Quantum Chemistry* **2011**, *111*, 3273–3279, DOI: doi:https://doi.org/10.1002/qua.23052.

(16) Lischka, H.; Nachtigallová, D.; Aquino, A. J. A.; Szalay, P. G.; Plasser, F.; Machado, F. B. C.; Barbatti, M. Multireference Approaches for Excited States of Molecules. *Chemical Reviews* **2018**, *118*, 7293–7361, DOI: doi:10.1021/acs.chemrev.8b00244, PMID: 30040389.

(17) Kossoski, F.; Barbatti, M. Nuclear ensemble approach with importance sampling. *Journal of chemical theory and computation* **2018**, *14*, 3173–3183.

(18) Barbatti, M.; Sen, K. Effects of different initial condition samplings on photodynamics and spectrum of pyrrole. *International Journal of Quantum Chemistry* **2016**, *116*, 762–771.

(19) Wigner, E. On the quantum correction for thermodynamic equilibrium. *Physical review* **1932**, *40*, 749.

(20) Prlj, A.; Marsili, E.; Hutton, L.; Hollas, D.; Shchepanovska, D.; Glowacki, D. R.; Slavíček, P.; Curchod, B. F. E. Calculating Photoabsorption Cross-Sections for Atmospheric Volatile Organic Compounds. *ACS Earth and Space Chemistry* **2022**, *6*, 207–217, DOI: doi:10.1021/acsearthspacechem.1c00355.

(21) Prlj, A.; Hollas, D.; Curchod, B. F. E. Deciphering the Influence of Ground-State Dis-

tributions on the Calculation of Photolysis Observables. *The Journal of Physical Chemistry A* **2023**, *127*, 7400–7409, DOI: doi:10.1021/acs.jpca.3c02333, PMID: 37556330.

(22) Ceriotti, M.; Bussi, G.; Parrinello, M. Langevin Equation with Colored Noise for Constant-Temperature Molecular Dynamics Simulations. *Phys. Rev. Lett.* **2009**, *102*, 020601, DOI: doi:10.1103/PhysRevLett.102.020601.

(23) Ceriotti, M.; Bussi, G.; Parrinello, M. Colored-Noise Thermostats à la Carte. *Journal of Chemical Theory and Computation* **2010**, *6*, 1170–1180, DOI: doi:10.1021/ct900563s.

(24) Qu, C.; Conte, R.; Houston, P. L.; Bowman, J. M. Full-dimensional potential energy surface for acetylacetone and tunneling splittings. *Physical Chemistry Chemical Physics* **2021**, *23*, 7758–7767.

(25) Lowrey, A.; George, C.; d'Antonio, P.; Karle, J. Structure of acetylacetone by electron diffraction. *Journal of the American Chemical Society* **1971**, *93*, 6399–6403.

(26) Andreassen, A.; Bauer, S. The structures of acetylacetone, trifluoroacetyl-acetone and trifluoroacetone. *Journal of Molecular Structure* **1972**, *12*, 381–403.

(27) Srinivasan, R.; Feenstra, J. S.; Park, S. T.; Xu, S.; Zewail, A. H. Direct determination of hydrogen-bonded structures in resonant and tautomeric reactions using ultrafast electron diffraction. *Journal of the American Chemical Society* **2004**, *126*, 2266–2267.

(28) Iijima, K.; Ohnogi, A.; Shibata, S. The molecular structure of acetylacetone as studied by gas-phase electron diffraction. *Journal of Molecular Structure* **1987**, *156*, 111–118.

(29) Caminati, W.; Grabow, J.-U. The C2v Structure of Enolic Acetylacetone. *Journal of the American Chemical Society* **2006**, *128*, 854–857, DOI: doi:10.1021/ja055333g, PMID: 16417375.

(30) Bauer, S.; Wilcox, C. On malonaldehyde and acetylacetone: are theory and experiment compatible? *Chemical physics letters* **1997**, *279*, 122–128.

(31) Sliznev, V.; Lapshina, S.; Girichev, G. Ab initio structure investigation of the enol forms of β-diketones RCOCH2COR (R= H, CH3, CF3). *Journal of Structural Chemistry* **2002**, *43*, 47–55.

(32) Matanović, I.; Došlić, N.; Mihalić, Z. Exploring the potential energy surface for proton transfer in acetylacetone. *Chemical physics* **2004**, *306*, 201–207.

(33) Matanović, I.; Došlić, N. Infrared spectroscopy of the intramolecular hydrogen bond in acethylacetone: A computational approach. *The Journal of Physical Chemistry A* **2005**, *109*, 4185–4194.

(34) Campomanes, P.; Menéndez, M. I.; Sordo, T. L. Resonance assisted hydrogen bonding and dynamic mechanism for crystal disorder in the enolic form of acetylacetone: a theoretical analysis. *Journal of Molecular Structure: THEOCHEM* **2005**, *713*, 59–63.

(35) Dannenberg, J.; Rios, R. Theoretical study of the enolic forms of acetylacetone. How strong is the hydrogen bond? *The Journal of Physical Chemistry* **1994**, *98*, 6714–6718.

(36) Qu, C.; Houston, P. L.; Conte, R.; Nandi, A.; Bowman, J. M. Breaking the coupled cluster barrier for machine-learned potentials of large molecules: The case of 15-atom acetylacetone. *The Journal of Physical Chemistry Letters* **2021**, *12*, 4902–4909.

(37) Käser, S.; Unke, O. T.; Meuwly, M. Reactive dynamics and spectroscopy of hydrogen transfer from neural network-based reactive potential energy surfaces. *New Journal of Physics* **2020**, *22*, 055002.
